# Investigation into the Thermal Response and Pharmacological Activity of Substituted Schiff Bases on α-Amylase and α-Glucosidase

**DOI:** 10.3390/antiox7090113

**Published:** 2018-08-28

**Authors:** Bamidele Joseph Okoli, Johannes Sekomeng Modise

**Affiliations:** Institute of Chemical and Biotechnology, Vaal University of Technology, Vanderbijlpark 1900, South Africa; joe@vut.ac.za

**Keywords:** antidiabetic, antimicrobial, antioxidant, azomethine, diffractogram, thermogram, Schiff bases

## Abstract

The emphasis of previous studies has targeted the development of insulin mimic with little attention given to the development of metabolic enzyme inhibitors. Our focus is to synthesise nine *o*-hydroxy and *p*-nitro-azomethine analogues, investigate their digestive enzyme inhibitory capacity, as well as the antioxidant and antimicrobial activities. The substituted Schiff bases were analysed using thermal gravimetric analyser (TGA), X-ray diffractometer (XRD), nuclear magnetic resonance spectroscopy (NMR), elemental analyser, and Fourier-transform infrared spectroscopy (FT-IR). Determination of synthetic yield revealed that the *o*-hydroxy analogues produced the highest yield of ≥77.1% compared to *p*-nitro and unsubstituted analogues. Spectra study showed the presence of azomethine stretching vibration at 1698–1613 cm^−1^, proton signals at δ 8.46–9.81, and carbon signals at δ 145.95–159.53 ppm. Investigation into the thermal property indicated an elevated melting point for the *o*-hydroxy analogue, compared to the *p*-nitro derivative which showed high stability to heat. There are similarities in crystalline structure with few unique patterns suggesting different substituent group. The antioxidant activities of the substituted analogues registered low half maximal inhibitory concentration (IC_50_), with exception to the ferric reducing power; indicating that the Schiff bases are weak siderophores. All nine Schiff bases were bacteriostatic or fungistatic at the screened concentrations; however, the nitro-substituted analogues have an enhanced activity with Minimum Inhibitory Concentration (MIC) values of 0.03–2.54 µM. Both *o*-hydroxy and *p*-nitro-substitution does not improve the antifungal activity of the compounds against *A. niger*. The *o*-hydroxyl and *p*-nitro Schiff base derivatives showed enhanced activity towards the inhibition of α-amylase and α-glucosidase by hydroxylation and glycosylation, respectively. Although, hydroxy derivatives of sulphonic acid derived Schiff base slightly decreased the activities on α-glucosidase and α-amylase. Our findings suggest that *p*-nitro substitution enhances the in vitro nonenzymatic activity while the *o*-hydroxy derivatives are good hydrolase inhibitors. Therefore, substituent modification can be used as an enhancement technique in designing novel pharmacophore.

## 1. Introduction

Diabetes is an ancient disease and one of the top five causes of death worldwide and remains a crippling global health problem of the twenty-first century [1,2]. It is a major degenerative disease characterised by acute hyperglycaemia, affecting approximately 15 million people and having associated complications such as gastroparesis, hypertension, ketoacidosis, hyperosmolar hyperglycemic nonketotic syndrome (HNS), and nephropathy [3]. Generally, diabetes is subdivided into type 1 (a condition in which the pancreas produces little or no insulin) [4], and type 2 (a condition that affects the way the body processes blood sugar) [5].

A recent study has linked microflora as the possible cause of one of the most prevalent conditions of our time: obesity-related Type 2 diabetes [6]. In a study by McArdle et al. (2013) [7] and Lee (2013) [8], Type 2 diabetes was associated with abnormal energy metabolism and low-level chronic inflammation of fatty tissues. Also, the prolonged exposure to pathogenic infection produced by microorganism causes symptoms of Type 2 diabetes, including insulin resistance, glucose intolerance, and systemic inflammation. Treatment of pathogenic infections caused by staph bacteria or neutralising the superantigens might have the potential for preventing or treating Type 2 diabetes. Bacterial superantigens are known to interact with fat cells and the immune system to cause chronic systemic inflammation, which may lead to insulin resistance and other symptoms characteristic of Type 2 diabetes.

Oxidative stress has also been known to play a pivotal role in the development of diabetes and diabetes-induced complications. Under a diabetic condition, oxidative stress causes a significant reduction in the antioxidant level of the cells, deactivates critical antiatherosclerotic enzymes, alters the structural functions of type IV collagen, and increase proteins glycation [9]. Oxidative stress has a significant effect on glucose transporters (SLC2A) and in insulin receptor activity [10]. Previous study has shown that radical scavengers reduce serum glucose status alleviating diabetes and preventing secondary complications [11]. Currently, diabetic condition is controlled either via insulin injection or synthetic drugs. On the negative side, insulin carries a high risk of hypoglycemic reactions when an excess dose of inulin is used and the fact that it causes weight gain in many patients. Hence, the continuing search for novel drugs possessing potent antidiabetic activity with very low median effective dose(ED_50_) with little or no side effects.

Schiff bases are well-known group of compounds as a result of their diverse biological activities; prepared by the condensation of aldehyde derivatives and aniline. This unique property is due to the presence of the imine or azomethine linkage and heteroatoms present [12,13]. Recent studies on the antidiabetic activity of Schiff bases and its complexes has also been evaluated in animal models; resulting in significant reduction of the blood glucose level and also altered biochemical parameters with improved glucose balance in rats with diabetic conditions [14].

The presence of electron donating or withdrawing substituents on the phenyl ring influence the properties of most compounds [15,16]. Free radical scavenging potentials of most biological scaffolds are enhanced by the introduction of hydroxyl and phenyl groups [17,18]. In therapeutics research, the nitro group has been introduced on prodrugs to reduce cytotoxicity and improve antimicrobial and antiprotozoal activities. It has been reported that the presence of aromatic or heteroaromatic nucleus in most compounds broaden the biological activity [19,20]. Substituents effect on the physicochemical properties of biological compounds, influence reactivities, conformation, and thermal properties. Few research works have been targeted on substitution–molecular property relationship, on the contrary studies on the effect of substituent on the biological and thermal properties of Schiff bases have been comparatively rare.

**Scheme 1:**
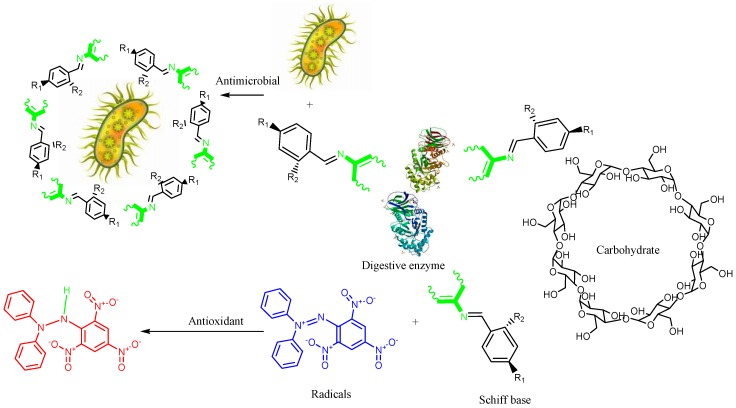
Gallery presentation of the study.

The primary focus of this paper is to analyse the influence of nitro and hydroxyl substituted Schiff bases on the in vitro inhibitory activity of α-amylase, and α-glucosidase as well as the antioxidant, antimicrobial, and thermal properties (Scheme 1). Overall, our data will assist researchers in predicting the physicochemical property in order to develop a pharmaceutically active molecule, to combat diabetic-related diseases worldwide.

## 2. Experimental Details

### 2.1. General

All chemicals were commercially available and are of analytical grade. Elemental compositions were estimated using CHNS elemental analyser (LECO Corp., Lakeview, MI, USA). Thermal properties were monitored on a thermogravimetric analyser (Perkin Elmer, Waltham, MA, USA). The functional groups were determined on FT-IR (PerkinElmer Spectrum 400, Waltham, MA, USA) scanned between 4000 and 400 cm^−1^. The X-ray diffractogram and wavelength maximum were investigated on a ZEISS Sigma 300 diffractometer (Oberkochen, Germany) and UV-visible spectrophotometer (Agilent Technologies Cary 60 UV-Vis, Santa Clara, CA, USA), respectively. Nuclear Magnetic Resonance spectra were recorded on VnmrJ3 400 MHz spectrometer (Agilent Technologies, Woodland, CA, USA). ^1^H-NMR and ^13^C-NMR chemical shifts are reported in ppm from TMS scale. Melting points were measured with a Stuart digital apparatus (Vernon Hills, IL, USA).

### 2.2. Synthesis

**Figure 1:**
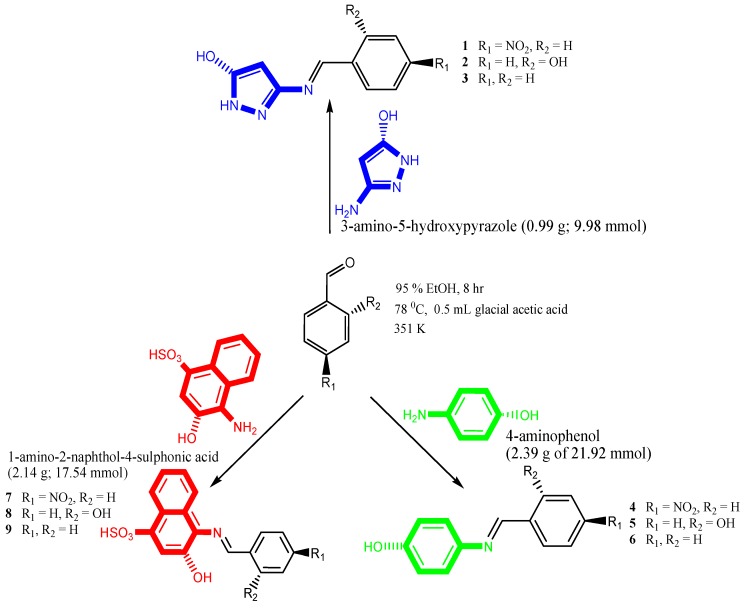
Synthetic route for compounds **1**–**9**.

Schiff bases were synthesised according to standard protocol [21], on a temperature regulated 6 plus carousel station equipped with a magnetic stirrer (Figure 1). Copies of the FT-IR and NMR spectra were presented as Supplementary Material for structural elucidation.

### 2.3. Electronic Absorption Studies

The absorbances of 0.4167 mg/mL Schiff bases (**1**–**9**) in dimethyl sulfoxide (DMSO) were measured on a UV-visible spectrophotometer. After which, the molar absorptivity coefficients were calculated from Lambert–Beer Equation (1):

(1) $$A\;=\;\varepsilon_{\lambda }bC$$

where *A* is the absorbance, *b* is the path length (1 cm), *λ* is the monochromatic wavelength, and $\varepsilon_{\lambda }$ is the molar absorption coefficient [22].

### 2.4. Antioxidant Assays

The antioxidant activities of Schiff bases (**1**–**9**) were determined by DPPH, ABTS^+^, H_2_O_2_, ferric reducing power assays, and compared with 3,4,5-trihydroxybenzoic acid (**10**), 5-(1,2-dihydroxyethyl)-3,4-dihydroxyfuran-2(5*H*)-one (**11**), and 2-(3,4-dihydroxyphenyl)-3,5,7-trihydroxy-4*H*-chromen-4-one (**12**) (as controls). Dilutions (125–1000 μM) of Schiff bases (**1**–**9**) and controls were prepared in DMSO.

#### 2.4.1. DPPH Radical Scavenging Activity

The scavenging activity of compounds (**1**–**12**) was evaluated by adding an ethanolic solution of DPPH (300 μL, 0.05 mM) and incubated for 30 min in the dark. After that, changes in the absorbance of the mixture were measured at 517 nm on a UV-Vis spectrophotometer [23]. The percentage radical scavenging activities (%RSA) and half maximal inhibitory concentration (IC_50_) of the compounds were estimated from Equation (2) and by linear regression analysis, respectively. All studies were carried out in triplicate.

(2) $$\%\;\mathrm{RSA}=\;100\lfloor \frac{\left(\mathrm{Abs}.\;\mathrm{of}\;\mathrm{DPPH}\;-\;\mathrm{Abs}.\;\mathrm{of}\;\mathrm{test}\;\mathrm{compound}\right)}{\left(\mathrm{Abs}.\;\mathrm{of}\;\mathrm{DPPH}\right)}\rfloor$$

#### 2.4.2. ABTS Radical Cation Decolourisation Assay

A mixture of ABTS (960.2 mg) and potassium persulphate (165.6 mg) was dissolved in 250 mL of distilled water to generate the blue-green ABTS^+^ solution. The ABTS solution was stored away in the dark at ambient temperature for 24 h to generate sufficient ABTS radical cation (ABTS^+^), then the absorbance was adjusted to 0.9547 with distilled water. Precisely 40 µL solution of the compounds were added to 3 mL ABTS^+^ solution and incubated at 298 K for 30 min in the dark. Changes in concentration of ABTS^+^ were measured at 734 nm on a spectrophotometer. The degree of ABTS^+^ decolourisation was a template for evaluating the percentage proton-donating potential (%PDP) of the test compounds [24]; estimated from Equation (3). The IC_50_ (μM) of the compounds were determined by linear regression analysis, and all analyses were carried out in triplicate.

(3) $$\%\;\mathrm{PDP}\;=\;100\lfloor \frac{\left(\mathrm{Abs}.{\;\mathrm{of}\;\mathrm{ABTS}\;}^{+}\;-\;\mathrm{Abs}.\;\mathrm{of}\;\mathrm{test}\;\mathrm{compound}\right)}{\left(\mathrm{Abs}.\;\mathrm{of}\;{\mathrm{ABTS}}^{+}\right)}\rfloor$$

#### 2.4.3. H_2_O_2_-Scavenging Activity

According to the methods of Ruch et al. [25], the H_2_O_2_ scavenging activity of compounds (**1**–**12**) was evaluated. To 100 µL aliquot of the test compounds was added 0.4 mL of 50 mM phosphate buffer followed by 0.6 mL of 2 mM H_2_O_2_ solution prepared in 50 mM phosphate buffer (pH 6.8). The absorbance of the mixture was measured at 230 nm, thereafter, the %H_2_O_2_ scavenging activity (Equation (4)) and IC_50_ (μM) of the compounds **1**–**12** was determined. All analyses were carried out in triplicate.

(4) $$\%\;H_{2}O_{2}\;-\;\mathrm{scavenging}\;\mathrm{activity}\;=\;100\lfloor \frac{\left(\mathrm{Abs}.{\;\mathrm{of}\;H}_{2}O_{2}\;-\;\mathrm{Abs}.\;\mathrm{of}\;\mathrm{test}\;\mathrm{compound}\right)}{\left(\mathrm{Abs}.\;\mathrm{of}\;H_{2}O_{2}\right)}\rfloor$$

#### 2.4.4. Reducing Power Assay

The ferric reducing activity was evaluated according to the method of Oyaizu [26], with slight modification. The reduction assay involved addition of 0.5 mL aliquot of compounds (**1**–**12**), 2 mL phosphate buffer (0.2 mole/L, pH 6.8) and 2 mL potassium ferricyanide (10 mg/mL)_._ Then, the mixture above was incubated at 318 K for 30 min followed by the introduction of 2 mL of trichloroacetic acid (100 mg/L). Thereafter, a 2-mL portion of the incubated mixtures was transferred into 2 mL of distilled water, and ferric chloride (0.4 mL, 0.1% *w*/*v*) and left to stand. After 10 min, the concentration of Fe^2+^ complex was measured at 700 nm and reported as the reducing power of compounds **1**–**12**.

### 2.5. Antimicrobial Studies

#### 2.5.1. Preparation of Resazurin-Based Indicator and Solution of Schiff Bases

Exactly 1.5 mg of sodium;10-oxido-7-oxophenoxazin-10-ium-3-olate was dissolved in 100 mL of distilled water, vortexed, filtered, sterilised, and stored at 4 °C for a maximum of 14 days before being applied as a growth indicator. 1000 μM stock solution of Schiff bases (**1**–**9**) was prepared in DMSO.

#### 2.5.2. Test Microorganisms

Fungi (*Aspergillus niger*, *Candida albicans*), Gram-positive bacteria (*Staphylococcus aureus*, *Enterococcus faecalis*), and Gram-negative bacteria (*Salmonella typhi*, *Escherichia coli*).

#### 2.5.3. Preparation of Standardised Inoculum

Colonies from a 24 h culture agar plate were collected with a loop and transferred to a tube containing lysogeny broth (LB). The various suspensions were incubated at 37 °C and the size adjusted to 1.5 × 10^6^ CFU/mL [27].

#### 2.5.4. Antimicrobial Assay of Schiff Bases **1**–**9**

The antimicrobial activity of Schiff bases (**1**–**9**), was evaluated using 96-well broth microdilution method as described by de Rapper et al. (2013) [28] and Akhalwaya et al. (2018) [29] with slight modifications. Columns 1–11 was filled 50 µL LB, followed by the addition of 50 µL of Schiff base to column 1. Thereafter, 50 µL of Schiff bases from column 1 was transferred by multichannel pipette to columns 2–10, resulting in 50 µL Schiff bases per well. Column 11 contained 100 µL of diluted standardised inoculum, while Column 12 contained 100 µL of LB as a control to monitor sterility. Then, 50 µL of the microorganism suspension was then added to columns 1–10 and the control column 11. The plates were incubated at 37 °C for 24 h, followed by the addition of 30 µL of growth indicator solution and further incubated for 2 h for the observation of colour change. On completion of incubation, the columns with blue indicator colour were reported as the MIC (µM) values. The columns with a concentration higher than the MIC value were plated on nutrient agar to determine the minimum bactericidal and fungicidal concentrations.

### 2.6. In Vitro Antidiabetic Activity

#### 2.6.1. α-Amylase Inhibitory Activity

The α-amylase inhibitory activity of Schiff bases (**1**–**9**) was evaluated by the method reported by Xiao et al. [30] with slight modifications. Substrate solution is prepared by dissolving 1.0 g of starch in 50 mL of 0.4 M NaOH and heated at 100 °C for 5 min. The substrate solution was adjusted to a neutral pH by the addition of 0.1 M HCl, cooled on crushed ice and made to 100 mL in a standard volumetric flask with distilled water. Various concentrations (2500 µM, 1000 µM, 750 µM, 500 µM, 250 µM, and 125 µM) of the Schiff bases were prepared in dimethyl sulphoxide-acetate buffer (pH 6.5). Using a microplate, 40 μL substrate and 20 μL Schiff base solution were mixed, then preincubated at 37 °C for 3 min. Thereafter, 20 μL of 50 μg/mL α-amylase solution was dispensed into each well, and further incubated for 15 min. The reaction was terminated by the addition of 80 μL HCl (0.1 M); followed by 200 μL of 1 mM iodine solution, and the absorbances measured at 650 nm. Inhibitory activity was calculated from Equation (5):

(5) $$\alpha \;-\;\mathrm{amylase}\;\mathrm{inhibition}\;\left(\%\right)\;=\;\left(\frac{A_{2}\;-\;A_{1}}{A_{4}\;-\;A_{3}}\right)100$$

where $A_{1}$ is the absorbance of Schiff base, starch, and α-amylase solution after incubation, $A_{2}$ is the absorbance of Schiff base and starch solution after incubation, $A_{3}$ is the absorbance of starch and amylase solution after incubation, and $A_{4}$ is the absorbance of a starch solution after incubation. Thereafter, the IC_50_ value was determined from the plot of % inhibition versus concentration. Acarbose was used as the positive control, and the assay was performed in triplicate.

#### 2.6.2. α-Glucosidase Inhibitory Activity

The inhibitory activity of Schiff bases (**1**–**9**) against α-glucosidase was assessed by the method previously described by Ranilla et al. (2010) [31]. Schiff bases concentrations (125–2500 μM) were prepared as in the α-amylase inhibitory assay above. Then, approximately 0.2 mL of Schiff base solution was mixed with 1.0 mL of 50 mM sodium phosphate buffer (pH 6.9) and 50 µL of α-glucosidase solution (2 U/mL). Thereafter, incubated at 37 °C for 10 min, followed by the addition of exactly 50 µL of 0.7 mM *p*-nitrophenyl-α-d-glucopyranoside solution in 50 mM sodium phosphate buffer (pH 6.9) and further incubated for 30 min at 37 °C. The reaction was terminated by the addition of 2.0 mL solution of 0.5 M Na_2_CO_3_. 0.3 mL aliquot of the above mixture was mixed with 4.7 mL of distilled water and the absorbance measured at 405 nm. Acarbose was equally used as a positive control. The percentage of enzyme inhibition was calculated from Equation (6) and plotted against concentration to determine the IC_50_.

(6) $$\alpha \;-\;\mathrm{glucosidase}\;\mathrm{inhibition}\;\left(\%\right)\;=\;\left(\frac{A_{e}\;-\;A_{SB}}{A_{e}}\right)100$$

where $A_{e}$ is the absorbance of the enzyme solution after incubation and $A_{SB}$ is the absorbance after incubation of Schiff bases solution. The assay was performed in triplicate.

## 3. Results

### 3.1. Synthesis and Spectra of Compound (***1**–**9***)

The synthesises were accomplished by the condensation of the ethanolic solution of aromatic amine and substituted benzaldehyde with either an electron withdrawing (–NO_2_) or electron donating (–OH) functionality. The condensation protocol revealed that the *o*-hydroxyl analogues gave the highest yield compared to the *p*-nitro and unsubstituted derivatives. In Table 1, the various yields, crystal colours, wavelength maximum ($\lambda_{max}^{*}$), and molar absorptivity coefficients ($\varepsilon_{max}^{*}$) of Schiff-bases (**1**–**9**) were recorded.

**Figure 2:**
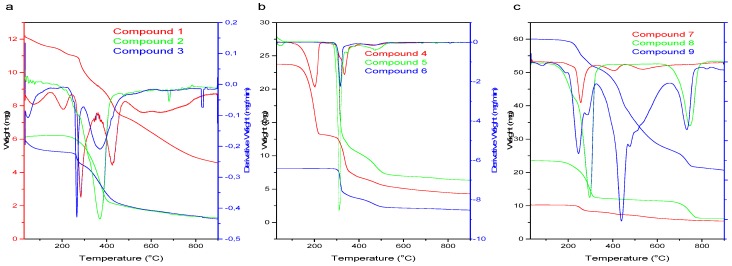
(**a**–**c**) Thermogravimetric and derivative thermal analysis plots of Schiff bases **1**–**9**.

The FT-IR spectra of Schiff bases (**1**–**9**), recorded in the mid-IR range 400–4000 cm^−1^ are presented in Table 2 and spectra provided as Supporting Information (Figures S1–S3) The thermogram of Schiff bases showed at least two endothermic peaks; the first corresponds to the removal of crystallising liquor at 80 °C. The second endothermic peak corresponds to the melting point of the Schiff bases, with the *p*-nitro-substituted Schiff bases (**1**, **4**, and **7**) showing some degree of depression in melting point (Figure 2a–c). All the *o*-hydroxy-substituted Schiff bases (**2**, **5**, and **8**) showed a slightly higher melting point, compared to the *p*-nitro-substituted Schiff bases (**1** and **4**) which recorded higher thermal stability compared to their corresponding *o*-hydroxy and unsubstituted Schiff bases.

### 3.2. Thermal Profile of Schiff Bases *(**1**–**9**)*

The thermograms of Schiff bases (**1**–**9**), obtained from the thermogravimetric analysis at 0 °C to 900 °C under a nitrogen atmosphere at a heating rate of 10 °C/min, are presented in Figure 2a–c. There were changes in thermal responses with substitution.

### 3.3. X-ray Diffraction

**Figure 3:**
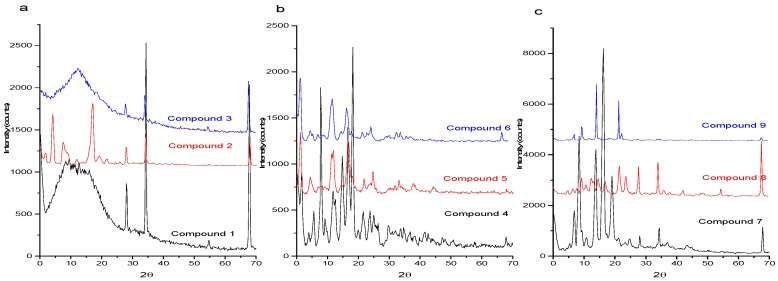
(**a**–**c**) Diffractogram of Schiff bases **1**–**9**.

The diffractogram presented in Figure 3a–c describes the specific chemistry and atomic arrangement of the Schiff bases (**1**–**9**). There were common peaks at 27.93°, 34.46°, and 67.59° for (**1**–**3**), 11.83°, 16.50°, and 67.72° for (**4**–**6**), and 28.03°, 34.38°, and 67.80° for (**7**–**9**); which confirmed the similarity in crystalline structure of the unsubstituted and corresponding substituted Schiff bases.

The average crystallite size (D), calculated from Scherrer formula (Equation (7)) and percentage change in crystallite size (Equation (8)) of the Schiff bases, were reported in Table 3.

(7) D=Kλ/βcosθ

where, *λ* is the wavelength (1.5406 $\overset{\cdot }{A}$), β is the integral height to width of the diffraction peak, and K is the equipment constant (0.94).

(8) $$\%\;\mathrm{change}\;\mathrm{in}\;\mathrm{crystallite}\;\mathrm{size}=100\left[\frac{\left(D_{\mathrm{USB}}-D_{\mathrm{SSB}}\right)}{D_{\mathrm{USB}}}\right]$$

where $D_{\mathrm{USB}}$ and $D_{\mathrm{SSB}}$ are crystallite sizes of unsubstituted and the corresponding substituted Schiff bases, respectively.

### 3.4. ^1^H and ^13^C NMR Spectra of Schiff Bases ***1**–**9***

The ^1^H and ^13^C-NMR spectra of Schiff bases (**1**–**9**) were determined in DMSO-d6 and characterization data for Schiff bases **1**–**9** are presented below while the copies of NMR spectra are included as Supplementary Materials (Supplementary file Figures S4–S21).

*3-(((4-nitrophenyl)methylidene)amino)-1H-pyrazol-5-ol* (**1**): ^1^H NMR (400 MHz, DMSO-*d*_6_) δ 9.13 (s, 1H), 8.35–8.28 (m, 2H), 8.19–8.11 (m, 2H), 6.34 (s, 1H). ^13^C NMR (100 MHz, DMSO-*d*_6_) δ 160.47, 156.85, 152.56, 149.35, 139.05, 129.41, 124.39, 85.93.; FT-IR (cm^−1^): 3620 (O–H), 1608 (C=C), 1531(N–O), 1328 (N–O) and 1697 (C=N); elemental analysis calc. for C_10_H_8_N_4_O_3_: %C, 51.73; %H, 3.47; %N, 24.13. Found: %C 52.80, %H 3.72, %N 26.10.

*3-(((2-hydroxyphenyl)methylidene)amino)-1H-pyrazol-5-ol* (**2**): ^1^H NMR (400 MHz) δ 9.81 (s, 1H), δ 7.44 (s, 1H), 7.03 (s, 1H), 6.16–6.04 (m, 3H), 5.82 (d, *J* = 10.7 Hz, 2H), 5.79–5.69 (m, 3H), 5.43–5.32 (m, 3H), 3.73 (s, 2H); ^13^C NMR (100 MHz, DMSO-*d*_6_) δ 163.06, 160.61, 160.07, 151.27, 132.61, 131.85, 121.13, 119.17, 116.94, 86.27; FT-IR (cm^−1^): 3460 (O–H), 3327 (N–H), 1698 (C=N), 1481–1604 (C=C); elemental analysis calc. for C_10_H_9_N_3_O_2_: %C, 59.11; %H, 4.46; %N, 20.68. Found: %C 57.39, %H 4.35, %N 21.92.

*3-((phenylmethylidene)amino)-1H-pyrazol-5-ol* (**3**): ^1^H NMR (400 MHz, DMSO-*d_6_*) δ 10.01 (s, 3H), 7.90 (s, 2H), 7.72 (s, 2H), 7.27 (s, 3H), 5.72 (s, 1H), 4.38 (s, 2H), 2.08 (s, 1H); ^13^C NMR (100 MHz, DMSO-*d_6_*) δ 160.07, 157.79, 152.62, 135.48, 130.48, 128.86, 128.62, 86.12; FT-IR (cm^−1^): 1684 (C=N), 1614 (C=C) and 1540 (N–H); elemental analysis calc. for C_10_H_9_N_3_O: %C, 64.16; %H, 4.85; %N, 22.45. Found: %C 65.18, %H 4.58, %N 23.89.

*4-(((4-nitrophenyl)methylidene)amino)phenol* (**4**): ^1^H NMR (400 MHz, DMSO-*d*_6_) δ 9.05 (s, 1H), 8.80 (s, 1H), 8.36–8.29 (m, 2H), 8.19–8.12 (m, 2H), 7.17–7.10 (m, 2H), 6.86–6.79 (m, 2H); ^13^C NMR (100 MHz, DMSO-*d*_6_) δ 159.53, 156.25, 149.03, 143.64, 139.10, 129.52, 124.51, 123.11, 116.69; FT-IR (cm^−1^): 3228 (O–H), 1695 (C=N), 1481–1608 (C=C) and 1429 (N–O); elemental analysis calc. for C_13_H_10_N_2_O_3_: %C, 64.46; %H, 4.16; %N, 11.56. Found: %C 65.96, %H 4.32, %N 11.08.

*4-(2-hydroxybenzylideneamino)phenol* (**5**): ^1^H NMR (400 MHz, DMSO-*d*_6_) δ 9.04 (s, 1H), 8.59 (s, 1H), 7.56–7.49 (m, 1H), 7.30 (td, *J* = 7.5, 1.5 Hz, 1H), 7.16–7.09 (m, 2H), 6.98 (td, *J* = 7.5, 1.5 Hz, 1H), 6.91–6.86 (m, 1H), 6.86–6.79 (m, 2H); ^13^C NMR (101 MHz, DMSO-*d*_6_) δ 146.95, 145.51, 128.66, 128.54, 127.36, 124.25, 123.83, 120.39, 116.90; FT-IR (cm^−1^): 3228 (O–H), 1656 (C=N), 1464–1612 (C=C); elemental analysis calc. for C_13_H_11_NO_2_: %C, 73.23; %H, 5.20; %N, 6.57. Found: %C 671.89, %H 5.09, %N 6.92.

*4-(benzylidene amino)phenol* (**6**): ^1^H NMR (400 MHz, DMSO-*d*_6_) δ 8.79 (d, *J* = 8.5 Hz, 4H), 7.84 (d, *J* = 6.0 Hz, 6H), 7.63–7.54 (m, 5H), 7.46–7.38 (m, 3H); ^13^C NMR (101 MHz, DMSO-*d*_6_) δ 146.56, 128.65, 128.41, 127.32, 124.30, 123.88, 120.47, 116.98; FT-IR (cm^−1^): 3232 (O–H), 1656 (C=N), 1408–1608 (C=C); elemental analysis calc. for C_13_H_11_NO: %C, 79.16; %H, 5.62; %N, 7.10. Found: %C 78.86, %H 5.77, %N 7.70.

*4-(4-nitrobenzylideneamino)-3-hydroxynaphthalene-1-sulphonic acid* (**7**): ^1^H NMR (400 MHz, DMSO-*d*_6_) δ 9.65 (s, 1H), 8.66 (s, 1H), 8.65 (dt, *J* = 7.6, 1.1 Hz, 1H), 8.30–8.22 (m, 3H), 8.13–8.06 (m, 2H), 7.80 (s, 1H), 7.57 (td, *J* = 7.5, 1.5 Hz, 1H), 7.40 (td, *J* = 7.5, 1.5 Hz, 1H); ^13^C NMR (101 MHz, DMSO-*d*_6_) δ 157.72, 155.21, 148.79, 142.58, 142.07, 129.55, 124.46, 123.64, 116.28; FT-IR (cm^−1^): 3428 (O–H), 1685 (C=N), 1508 (N–O), 1578–1608 (C=C); elemental analysis calc. for C_17_H_12_N_2_O_6_S: %C, 54.84; %H, 3.25; %N, 7.52; %S, 8.61. Found: %C 55.12, %H 3.56, %N 7.46, %S 8.08.

*4-(2-hydroxybenzylideneamino)-3-hydroxynaphthalene-1-sulphonic acid* (**8**): ^1^H NMR (400 MHz, DMSO-*d*_6_) δ 9.46 (s, 1H), 8.71–8.62 (m, 2H), 8.24 (dd, *J* = 7.5, 1.5 Hz, 1H), 7.80 (s, 1H), 7.59–7.50 (m, 2H), 7.43 (td, *J* = 7.5, 1.5 Hz, 1H), 7.30 (td, *J* = 7.5, 1.5 Hz, 1H), 6.97–6.85 (m, 2H); ^13^C NMR (100 MHz, DMSO-*d*_6_) δ 163.46, 160.20, 151.97, 143.99, 140.96, 132.84, 130.91, 130.83, 127.76, 127.22, 127.05, 125.51, 122.59, 121.23, 121.01, 117.04, 109.62; FT-IR (cm^−1^): 3341(O–H), 1621 (C=N), 1433–1604 (C=C); elemental analysis calc. for C_17_H_13_NO_5_S: %C, 59.47; %H, 3.82; %N, 4.08; %S, 9.34. Found: %C 59.10, %H 3.57, %N 4.61, %S 9.74.

*4-(benzylidene amino)-3-hydroxynaphthalene-1-sulphonic acid(**9**)*: ^1^H NMR (400 MHz, DMSO-*d*_6_) δ 8.60 (s, 1H), 8.46 (d, *J* = 0.5 Hz, 1H), 7.98–7.88 (m, 2H), 7.54–7.44 (m, 3H), 7.26–7.17 (m, 2H), 6.92–6.83 (m, 2H); ^13^C NMR (101 MHz, Acetone-*d*_6_) δ 157.08, 156.29, 143.74, 137.01, 130.70, 128.62, 128.33, 122.37, 115.68; FT-IR (cm^−1^): 3577 (O–H), 1616 (C=N), 1449–1690 (C=C); elemental analysis calc. for C_17_H_13_NO_4_S: %C, 62.37; H, 4.00; N, 4.28; S, 9.80. Found: %C 62.86, %H 4.77, %N 3.86, %S 9.70.

### 3.5. Antioxidant Activity of Schiff Bases ***1**–**9***

In this study, the scavenging activity of the Schiff bases (**1**–**9**) and controls (**10**–**12**) was expressed as the IC_50_ (µM) measured at the characteristic wavelength maximum $\lambda_{\max}$ for each radical assay and presented in Table 4.

**Figure 4:**
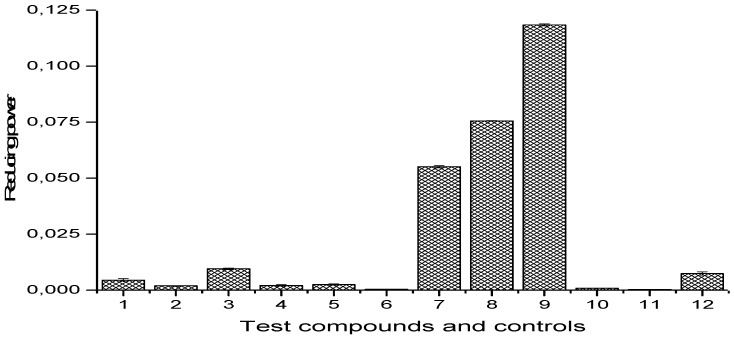
Reducing power of Schiff bases **1**–**9** and controls **10**–**12** measured at 700 nm (concentration of 200 µM). Each value is the mean ± SD.

The reducing powers were reported as a function of their absorbance and presented in Figure 4. In this assay, the presence of antioxidants initiates the reduction of the Fe^3+^/ferricyanide complex to the ferrous form. Therefore, measuring the formation of Perl’s Prussian blue at 700 nm is a measure of the Fe^2+^ concentration.

### 3.6. Antimicrobial Activity

**Figure 5:**
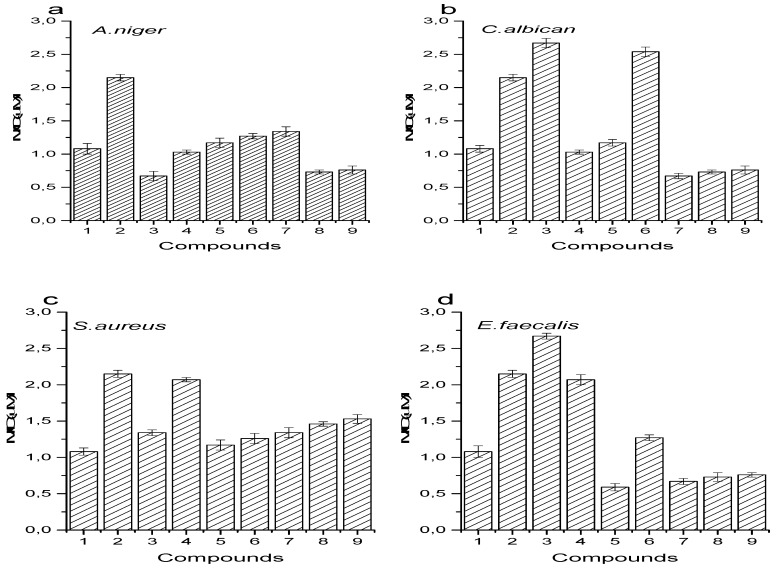

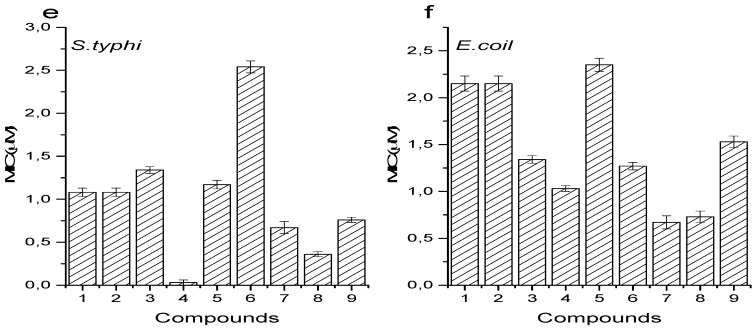
(**a**–**f**) Minimum Inhibitory Concentration (µM) of Schiff bases **1**–**9**.

The minimum inhibitory concentrations of Schiff bases **1**–**9** are presented in Figure 5a–f. The most potent antifungal Schiff bases are (**3**) and (**7**) with MIC values of 0.67 µM. However, the activity of (**1**–**3**) against *A*. *niger* was not influenced by either *o*-hydroxyl or *p*-nitro substitution while the anti-candida activity of the *p*-nitro-substituted Schiff base (**3**) is significant. Conversely, there was no significant difference (*p >* 0.05) in the anti-*candida* activity of Schiff bases (**7**–**9**)**.** The anti-candida and niger activities of (**4**–**6**) were influenced by substituent type, with the nitro-derivative registering a significant activity against the tested fungi (Figure 5a,b). Generally, the antifungal activity of the Schiff bases was enhanced by substitution, with the *p*-nitro derivative exhibiting potent activity in comparison with the o-hydroxy derivative.

The bactericidal (MBC) and fungicidal (MFC) concentrations of the Schiff bases (**1**–**9**), were evaluated by selecting concentrations higher than the MICs and the results are presented in Table 5. The observed MBC and MFC showed that the Schiff bases were not all bactericidal and fungicidal on *p*-nitro or *o*-hydroxy substitution at the tested concentrations.

### 3.7. In Vitro α-Glucosidase and α-Amylase Inhibitory Activities

The enzymatic inhibitory effect of Schiff bases (**1**–**9**), were evaluated in comparison with an antidiabetic control (acarbose). The inhibitory activities of the studied compounds were all influenced to some degrees by substituent type and are reported in Table 6.

## 4. Discussion

### 4.1. Synthesis and Spectra of Compound ***1**–**9***

The inductive effect of the hydroxyl group was a significant factor, in regulating the yield of this Schiff base. Thereby the inductive effect of the hydroxyl group increases the electron density around the carbonyl group, and initiated an uncomplicated condensation reaction; hence, the high yield of hydroxyl derivatives (**2**, **5**, and **8**) compared to the *p*-nitro and unsubstituted derivatives. Conversely, the nitro group on derivatives **1**, **3**, and **7** reduces the electron density within the ring and around the carbonyl group hence, the observed low yields.

The wavelength maximum ($\lambda_{max}^{*}$) recorded for Schiff bases (1–9), were between 321 and 498 nm; associated with π→π* and n→π* transitions of the aromatic ring and azomethine group, respectively. The transition agrees with the molar absorptivity ($\varepsilon \;>10^{4}{\mathrm{Lmol}}^{-1}{\mathrm{cm}}^{-1}$) corresponding to symmetry allowed transitions, hence, the strong intensity of transition. The *p*-nitro and *o*-hydroxyl substitutions caused a bathochromic shift in the spectra of Schiff bases **1** and **7** and **2**, **5**, and **8**, respectively. However, a hypochromic shift was observed in spectra of *p*-nitro derivative (**4**), due to the opposing inductive property of the nitro and hydroxy groups around the azomethine unit.

The difference in the intensity of the vibrational bands indicated the presence of Schiff bases with a different dipole moment. Peaks at 1698–1613 cm^−1^ confirmed the presence of azomethine (–C=N–) stretching vibration [32], which was observed to be slightly shifted to a longer wavenumber. There was a significant shift to a longer wavenumber when a nitro group is introduced compared to a hydroxyl. Hence, the shift to a longer wavenumber is due to the large vibrational energy imposed by the negative inductive effect of the electron withdrawing group. Other vibrational bands at 3567–3219 cm^−1^ and 1575–1507 cm^−1^ were assigned to –O–H and –C–H stretching vibrations of the Schiff bases, respectively.

### 4.2. Thermal Profile and Crystal Sizes of Schiff Bases (***1**–**9***)

The high thermal response of the Schiff bases has implications in their biochemical and environmental parameters due to the relationship between temperature and solubility, which plays a critical factor in the design of drug solubility [33,34,35]. Therefore, such depression in the melting point effectively means an increase in biological fluid solubility and decrease aqueous solubility, according to a study by Abramowitz et al. (1990) [36].

The diffractogram produced a unique diffraction pattern for each Schiff bases; suggesting different substitution patterns. Study of the *o*-hydroxy-derivative (**2**) revealed a smaller nanocrystalline size indicating a homogeneous crystal composition compared to the broader peaks of the unsubstituted (**3**) and *p*-nitro derivative (**1**) derivatives. Nitro or hydroxy substitution on derivatives of **6** and **9** showed a decrease in the peak intensity, with a corresponding symmetrical crystalline compound. The ordered arrangement of atoms or molecules in a crystalline structure is associated with the crystallite size, which directly influences the properties such as the antibacterial [37] and crystal solubility [33]. There was no consistent trend in the degree of crystallite growth with substituent types. However, *p*-nitro substitution caused a significant change in crystallite sizes compared to the *o*-hydroxy derivatives. This is consistent with the observed decrease in the melting point of the nitro derivatives related to the solubility and on the effect electron withdrawing substituents on the supramolecular packings and average crystallite sizes of molecule [38].

### 4.3. ^1^H and ^13^C NMR Spectra of Schiff Bases ***1**–**9***

^1^H NMR spectrum of Schiff bases (**1**–**3**) showed aromatic signals at δ = (8.33–6.06) ppm, a clear azomethine proton signal at δ = (9.81–9.09) ppm, pyrazole proton signals at δ = (6.48–5.83) ppm, and the *o*-hydroxy proton of Schiff base **2** was registered at δ = 5.75 ppm (Supplementary file Figures S4, S6 and S8). The ^13^C-NMR spectrum showed pyrazole carbon signals at δ = (160.61–156.85) and aromatic carbon signals δ = (151.27–116.94), while the *o*-hydroxy carbon signal of Schiff base **2** was recorded at δ = 130.06 ppm due to the deshielding influence of the hydroxy group (Supplementary files Figures S5, S7 and S9). On the ^1^H-NMR spectrum of Schiff bases (**4**–**6**), aromatic multiplet signals were observed at δ = (8.78–6.81) ppm, azomethine proton signals at δ = (8.80–8.59) ppm, and hydroxy proton signals at δ = (11.06–9.04) ppm (Supplementary files Figures S10, S12 and S14). ^13^C-NMR spectrum showed aromatic carbon signals at δ = (156.25–111.76) ppm, azomethine carbon at δ = (159.53–145.95) ppm, and phenolic proton signals at δ = (149.03–128.66) ppm (Supplementary files Figures S11, S13 and S15). Finally, the ^1^H-NMR spectrum of compounds (**7**–**9**) showed clear phenolic proton and azomethine singlet at δ = (9.65–8.60) and δ = (8.46–8.69) ppm, respectively. The aromatic multiplet peaks were observed at δ = (8.46–6.87) ppm (Supplementary file Figures S16, S18 and S20), with the corresponding aromatic carbon signals at δ = (143.74–109.62) ppm. The azomethine and phenoxy carbon signals resonated at δ = (157.72–151.97) and δ = (156.29–116.28) ppm, respectively (Supplementary file Figures S17, S19 and S21). The presence of azomethine proton and a carbon signals were in concord with the reports from various literature confirming the successful synthesis of Schiff bases **1**–**9** [39,40].

### 4.4. Antioxidant Activity of Schiff Bases ***1**–**9***

Antioxidants are known to quench free radicals by producing stable substituted analogous of the radicals either by donation of hydrogen atoms or electron pairs [41]. Hence, the more rapidly the radical is scavenged, the more potent the antioxidant capacity of the Schiff base. The scavenging potential for the Schiff bases increased with the introduction of a substituent at either the *para* or *ortho* position.

The *p*-nitro derivatives (**1**) and (**7**), significantly scavenged the radical formed by DPPH, ABTS, and H_2_O_2_ compared to the other Schiff bases (**2** and **3**) and (**8** and **9**), respectively. On the other hand, the *o*-hydroxy Schiff base (**5**) is a more potent radical scavenger than the derivates (**4** and **5**). However, a close examination of the molecular structure of the potent *p*-nitro Schiff base derivative (**1**) and (**7**), suggest that in addition to the hydroxyl group present on the side ring of the Schiff bases, the electron withdrawing group (–NO_2_) initiates an electron donation process which quenches the radicals at an IC_50_ values of ≥290 ± 9 µM and ≥50 ± 5 µM, respectively. The nitro group decreases the electron density in the ring increasing the ease at which the lone pair of electron and acidic proton are donated towards the quenching of the radicals. In other words, the *p*-nitro Schiff base derivatives (**1**) and (**7**) scavenges active radicals by both hydrogen and electron donations, resulting in the very low IC_50_ values. The *o*-hydroxyl derivative (**5**) inhibited 50% of the DPPH, ABTS, and H_2_O_2_ radicals at concentrations of 260 ± 5, 110 ± 5, and 320 ± 6 µM, respectively; resulting from the mono-scavenging mechanism of proton donation. The presence of an additional hydroxyl group on Schiff base (**5**) helps to stabilise the phenoxy ion compared to Schiff bases (**4**) and (**6**) and shifting the equilibrium to the formation of the more stable bi-phenoxy ion, increasing the ease of acidic proton denotation. Considering, the weak acidity of phenolic hydrogen, the propensity to donate a proton into the medium will be very poor in comparison to *p*-nitro Schiff base derivatives (**1**) and (**7**). The *p*-nitro or *o*-hydroxy Schiff base substitution enhances the radical scavenging activity compared to the unsubstituted Schiff bases (**3**, **6**, and **9**) and controls.

Further, the effect of substitutions on the reducing power of the Schiff bases at a concentration of 200 µM was evaluated. Although, the reducing power of Schiff bases **1**–**6**, was not significantly influenced by substitution, most likely to the weak proton or electron donating capacity. Hence, the ability to donate a proton or release electron into the aqueous medium is impaired even in the presence of a substituent. The reducing power of Schiff bases **7**–**9** responded to substituent type and are significantly more potent than the controls. The *o*-hydroxy (**7**) and *p*-nitro (**8**) derivatives decreased the proton donating capacity of unsubstituted Schiff base (**9**) and significantly altered the reducing power [42]. This observation underlines the negative impact of substitution on the azomethine-type compound in reversing the ferric ion to the biologically useful ferrous form. Hence, the inclusion of substituent on Schiff bases (**1**–**9**) makes them poor siderophores; due to their weak capacity to reduce Fe^3+^/ferricyanide complex to the ferrous form.

### 4.5. Antimicrobial Activity

The potent antifungal activity of *p*-nitro derivative may be due to the capacity to influence the electronic characteristic and lipophilic property of Schiff bases (**1**, **4**, and **7**). Gram-negative organisms were more sensitive to Schiff bases (**1**–**9**) with MIC values of 0.03–2.35 µM (Figure 5c,d) in contrast to Gram-positive organisms (MIC values of 0.36–2.54 µM) (Figure 5e,f). However, Schiff bases (**7**–**9**) were significantly potent (*p* < 0.05) against all the test bacteria compared to the other Schiff bases (**1**–**6**).

The *p*-nitro Schiff base (**7**) was very potent against S. *aureus*, *E. coli*, and *E. faecalis* relative to the *o*-hydroxy derivative (**8**) which inhibited only the growth of *S. typhi*. It was observed that *p*-nitro and *o*-hydroxy substitution on Schiff bases with a pyrazole side chain did not impact the antibacterial activity compared to Schiff bases (**4**–**6**) and (**7**–**9**). Consequently, the antibacterial activity of Schiff bases (**1**–**9**), is dependent on the complexity of the structural backbone, electronic distribution, and substitution type. These properties influence the capacity of Schiff bases in breaching the thin peptidoglycan layer and the outer lipopolysaccharides membrane on the cell wall of the Gram-positive organism. However, Schiff bases (**7**–**9**) were fungicidal against *A. niger* and *C. albicans* with no bactericidal effect at the tested concentration against *S. aureus*, *E. faecalis*, and *S. typhi*. On the contrary, all the Schiff bases were bactericidal towards *E. coli* with the most effective being Schiff bases (**7**–**9**) with MBC values of 1.34–3.06 µM. Specific substitution types impose not only a bacteriostatic or fungistatic property on the Schiff bases but can also induce some bactericidal and fungicidal effect.

### 4.6. In Vitro α-Glucosidase and α-Amylase Inhibitory Activities

The degree at which both α-amylase and α-glucosidase is inhibited with substitution is dependent on the structural backbone of the Schiff base. Substitution of the Schiff bases by either the hydroxy or nitro group both enhanced enzymatic inhibition to various extent compared to their unsubstituted derivatives. The inhibition potential of the tested compounds showed that Schiff bases (**1**–**3**) were the most effective inhibitor against both α-amylase and α-glucosidase with IC_50_ values of $1.20\times 10^{2}\pm 0.51$ to $5.60\times 10^{2}\pm 0.82$ and $0.91\times 10^{2}\pm 0.09$ to $4.20\times 10^{2}\pm 0.36$ µM, respectively. The *o*-hydroxyl derivative (**2**, **5**, and **8**) showed very low IC_50_ values towards the inhibition of α-amylase and α-glucosidase and this is most likely due to the hydroxylation reaction. Similarly, the inhibitory activities of the *p*-nitro Schiff bases (**7**–**9**) showed enhanced activity against the digestive enzymes mostly by glycosylation of the functional unit compared to the unsubstituted derivatives [43]. The only exception was the reduced inhibitory activity of Schiff base (**1**) against α-amylase which might be due to its stereochemical configuration.

Schiff bases (**7**–**9**) showed the least inhibitory activity (IC_50_ values 4.20 × 10^2^ ± 0.34 to 9.61 × 10^2^ ± 0.84), as a result of the presence of a sulphonic acid moiety which disrupted the optimal operational pH of the enzymes as reported to in the study of Ahmed et al. (2007) [44]. There was no significant difference (*p* > 0.05) in the inhibition of both α-amylase, and α-glucosidase by Schiff bases (**4**–**6**) with IC_50_ values of $13.77\times 10^{2}\pm \;0.17$ to $14.14\times 10^{2}\pm \;0.17$ and $13.83\times 10^{2}\pm 12.53$ to $14.18\times 10^{2}\pm 17.34$ µM, respectively. Our results suggest that the digestion of starch and disaccharides to absorbable sugars, by α-glucosidase and α-amylase can be delayed by the aid hydroxylated and nitrated Schiff bases. Also, it was observed that Schiff bases (**1**–**9**) were more potent against α-amylase relative to α-glucosidase.

## 5. Statistical Analysis

Statistical analysis was carried out with Origin Pro software (Origin Lab Corporation, Northampton, MA, USA), and results are expressed as means ± standard deviation.

## 6. Conclusions

Hydroxyl and nitro derivatives of *3*-((phenyl methylidene) amino)-1*H*-pyrazol-5-ol (**3**), 4-(benzylidene amino) phenol (**6**), and 4-(benzylidene amino)-3-hydroxynaphthalene-1-sulphonic acid (**9**); were synthesised by template reaction. The azomethine, hydroxyl, and aromatic signals were confirmed on the FT-IR and NMR, while the response to substituent variations was reflected as a shift in wavelength, changes in thermal properties, and crystalline sizes measured on the UV-Vis., TGA, and XRD, respectively. Our investigation further revealed that hydroxylation and nitration could enhance specific biological activities of these Schiff bases. The *p*-nitro or *o*-hydroxy substitution of Schiff bases enhanced the antiradical activity compared to the unsubstituted Schiff bases but had weak ferric reducing property except for 4-(benzylidene amino)-3-hydroxynaphthalene-1-sulphonic acid derivatives. Both derivatives of 4-(benzylidene amino)-3-hydroxynaphthalene-1-sulphonic acid were potent fungicidal compounds while Schiff bases of (**3** and **6**) showed improved the bacteriostatic and fungistatic activities. Also, the conversion of starch and disaccharides to absorbable monosaccharides, by hydrolase can significantly be inhibited by hydroxylation and glycosylation; hence, potentially lowering the blood-glucose level. Future work will focus on profiling the cytotoxic property, mitochondrial integrity of the cells in response to nitration and hydroxylation of the Schiff bases; hence, providing more data for in vivo study.

## Figures and Tables

**Table 1:** Physio-parameters and UV-Visible data of Schiff bases (**1**–**9**).

Schiff Bases	M.F	M.pt (°C)	Yield %	Nature	$\lambda_{max}^{*}\;\left(\mathrm{nm}\right)$	$\varepsilon_{max}^{*}\;\times 10^{4}\;\left(L\;{\mathrm{mol}}^{-1}\;{\mathrm{cm}}^{-1}\right)$
1	232.07	285	61.8	Regatta	393	
2	232.06	370	77.1	Mandarin	470	
3	187.07	266	63.8	Peach Puff	321	0.8236
4	242.07	202	27.3	Silver	435	2.5340
5	213.08	305	95.6	Light gray	498	
6	197.08	302	39.7	Grey	472	2.8978
7	372.04	200	57.3	Orange	455	5.3109
8	343.05	296	93.7	Dirty green	397	2.6220
9	327.06	246	73.1	Chocolate	394	2.3770

* Wavelength maximum.

**Table 2:** The FT-IR spectral data of substituted Schiff bases (**1**–**9**).

Schiff Bases	FT-IR (cm^−1^)				
	υO−H	υC=N	υO−N	υC=C	υN=H
**1**	3352	1698	1508	1529	3590
**2**	3450	1698	-	1526	3338
**3**	3450	1690	-	1575	3660
**4**	3219	1660	1347	1521	-
**5**	3232	1655	-	1432	-
**6**	3236	1653	-	1533	-
**7**	3437	1689	1502	1575	-
**8**	3337	1613	-	1514	-
**9**	3567	1614	-	1507	-

**Table 3:** Crystallite size (nm) of Schiff base **1**–**9**.

Schiff Bases	1	2	3	4	5	6	7	8	9
**Crystallite size (nm)**	13.01	10.33	10.25	11.11	6.19	6.67	13.13	18.55	22.62
**% change in crystallite size**	−26.93	−0.78	−	−66.57	8.00	−	41.95	17.99	−

**Table 4:** Scavenging activity of Compounds **1**–**12**.

	Antioxidant Assay		
	DPPH	ABTS	H_2_O_2_-Scavenging
Compounds	IC_50_ (µM)	IC_50_ (µM)	IC_50_ (µM)
**1**	290 ± 9	410 ± 6	350 ± 5
**2**	390 ± 5	510 ± 5	410 ± 6
**3**	360 ± 5	450 ± 7	410 ± 8
**4**	290 ± 6	370 ± 6	370 ± 7
**5**	260 ± 5	110 ± 5	320 ± 6
**6**	380 ± 2	260 ± 6	380 ± 5
**7**	200 ± 0	50 ± 5	160 ± 90
**8**	210 ± 5	140 ± 5	240 ± 17
**9**	220 ± 6	50 ± 4	190 ± 11
**10**	420 ± 3	580 ± 7	460 ± 11
**11**	350 ± 5	530 ± 6	440 ± 7
**12**	240 ± 3	270 ± 8	270 ± 10

Mean value ± SD (*n* = 3).

**Table 5:** Minimum bactericidal (MBC) and fungicidal concentrations (MFC) of Schiff bases.

Compounds	MBC (µM)				MFC (µM)	
	*S. aureus*	*E. faecalis*	*E. coli*	*S. typhi*	*A. niger*	*C. albicans*
**1**	4.31	4.31	4.31	2.15	>4.31 *	>4.31 *
**2**	4.31	>4.31 *	4.31	>4.31 *	>4.31 *	>4.31 *
**3**	>5.35 *	>5.35 *	2.67	>5.35 *	>5.35 *	>5.35 *
**4**	4.31	>4.31 *	2.07	>4.31 *	>4.31 *	>4.31 *
**5**	>4.69 *	4.69	4.69	>4.69 *	>4.69 *	>4.69 *
**6**	>5.07 *	>5.07 *	2.54	>5.07 *	>5.07 *	>5.07 *
**7**	>2.69 *	>2.69 *	1.34	>2.69 *	2.69	1.53
**8**	>2.92 *	>2.92 *	1.46	>2.92 *	1.46	1.46
**9**	>3.06 *	>3.06 *	3.06	>3.06 *	1.53	1.53

* No bactericidal or fungicidal effect at the evaluated concentration. Mean value ± SD (*n* = 3).

**Table 6:** α-glucosidase and α-amylase inhibition activity of Schiff bases **1**–**9**.

Schiff Bases	α-Glucosidase	α-Amylase
**1**	5.40± 0.44	4.20 ± 0.36
**2**	1.20 ± 0.51	0.91 ± 0.09
**3**	5.60 ± 0.82	2.00 ± 0.74
**4**	13.94 ± 0.91	13.77 ± 0.17
**5**	13.83 ± 12.5	13.83 ± 0.84
**6**	14.18 ± 17.3	14.14 ± 0.17
**7**	9.61 ± 0.84	7.30 ± 0.16
**8**	5.00 ± 0.82	4.20 ± 0.34
**9**	6.60 ± 0.97	5.61 ± 0.42
Acarbose	8.33 ± 7.20	6.80 ± 1.25

Means $\times 10^{2}$ ± SD (IC_50_ (µM)) from at least three experiments.

## Supplementary Materials

The following are available online at http://www.mdpi.com/2076-3921/7/9/113/s1, including 1D NMR, and FT-IR spectra of Schiff bases **1**–**9**.
