# Supplementary figures and images for: Investigation into the Thermal Response and Pharmacological Activity of Substituted Schiff Bases on α-Amylase and α-Glucosidase
Source: Antioxidants (Basel). 2018 Aug 28;7(9):113. doi: 10.3390/antiox7090113 (PMC6162693; doi:10.3390/antiox7090113)

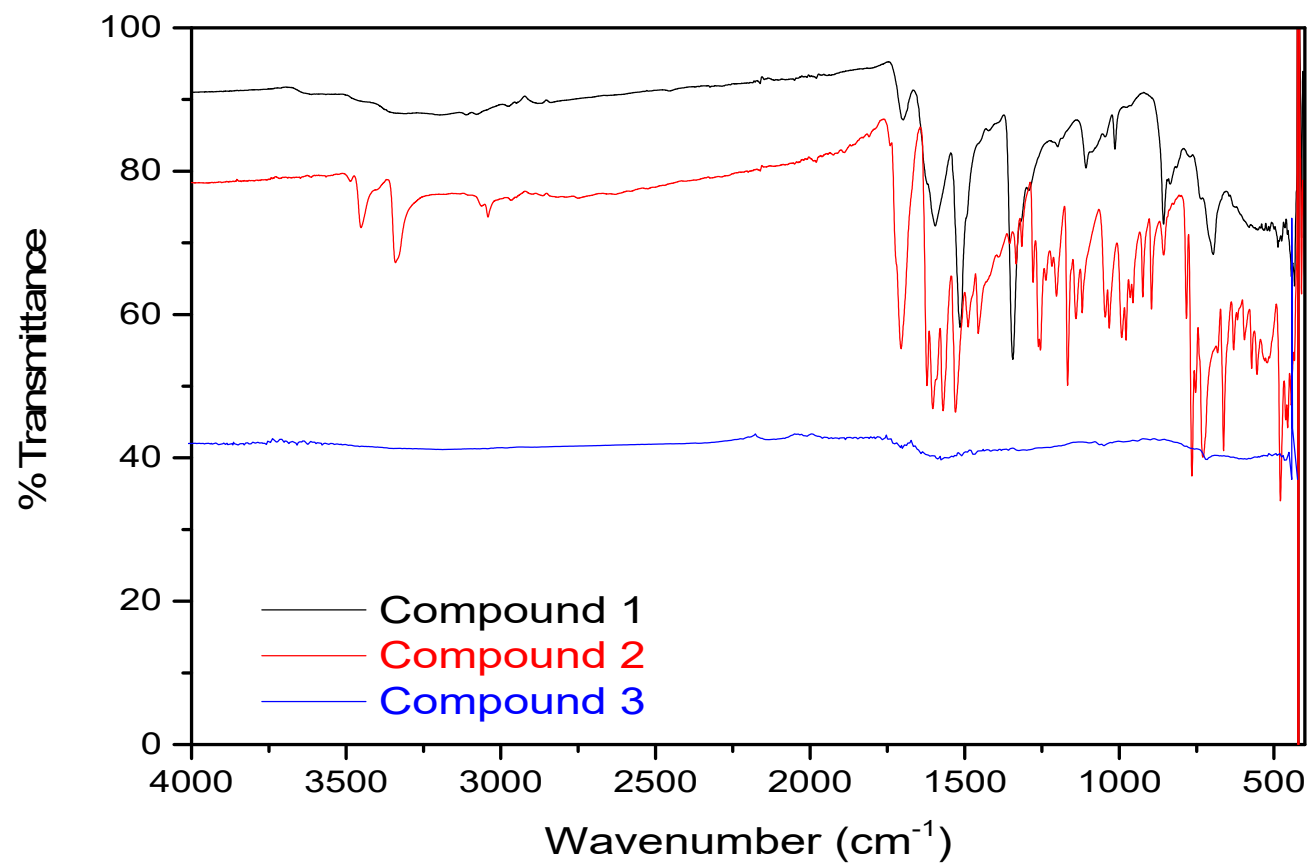

**Figure S1:** FT-IR spectrum of Schiff bases **1-3**

Supplement: Supplementary file 1 [file antioxidants-07-00113-s001.zip › Figure S1.pdf]

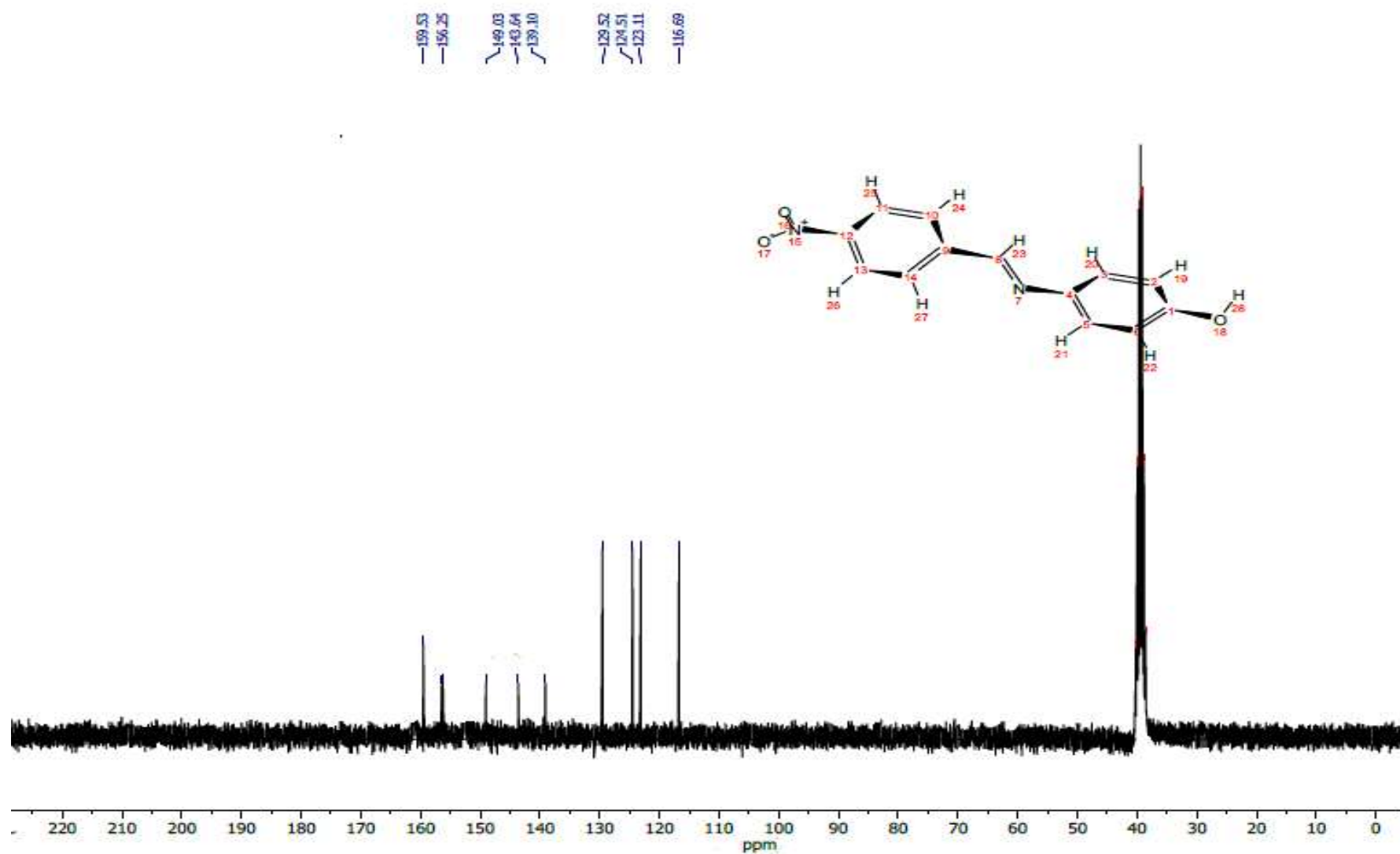

Figure S11:  $^{13}\text{C}$  NMR of Schiff base 4

Supplement: Supplementary file 1 [file antioxidants-07-00113-s001.zip › Figure S11.pdf]

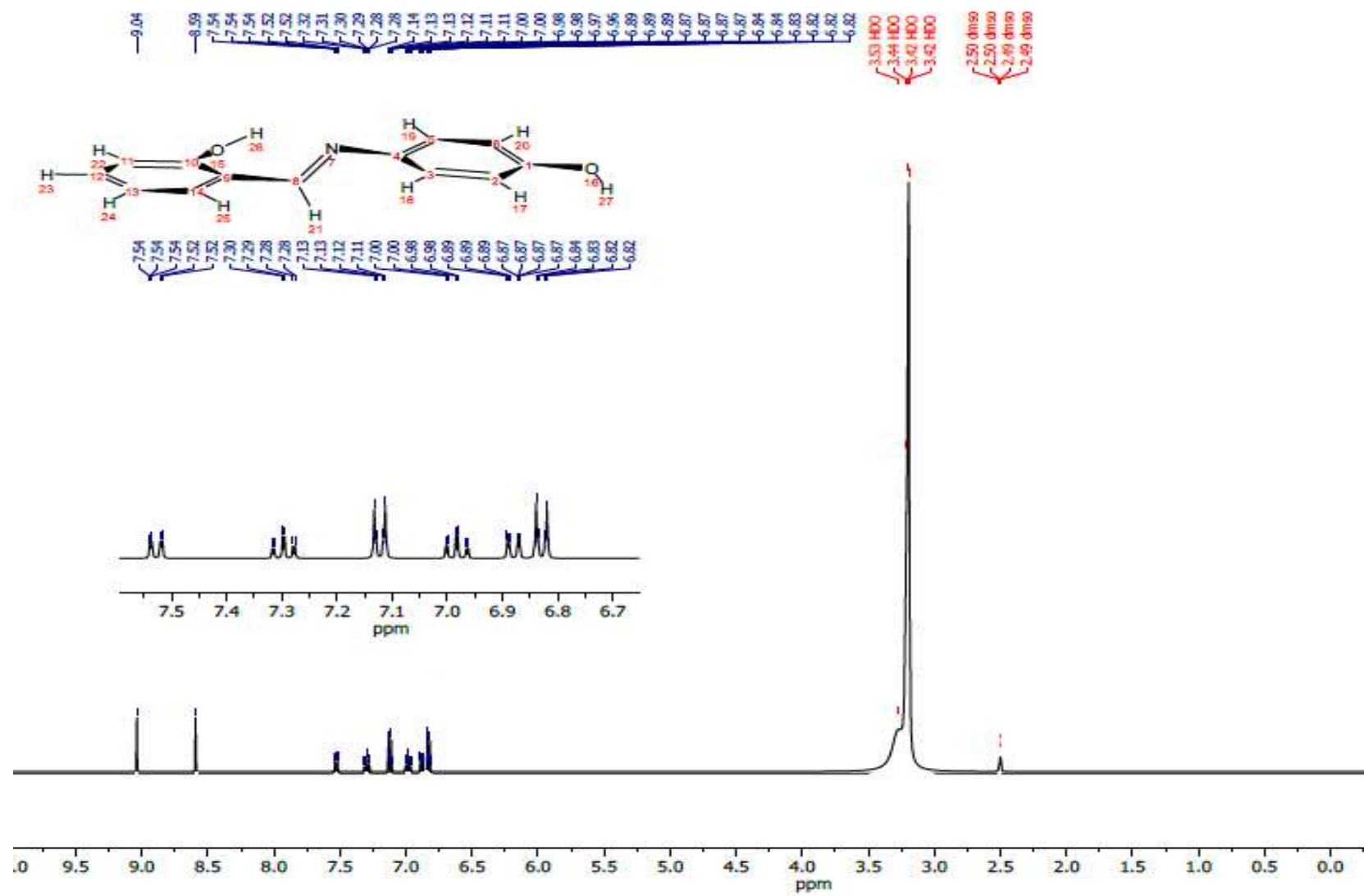

Figure S12:  $^1\text{H}$  NMR of Schiff base 5

Supplement: Supplementary file 1 [file antioxidants-07-00113-s001.zip › Figure S12.pdf]

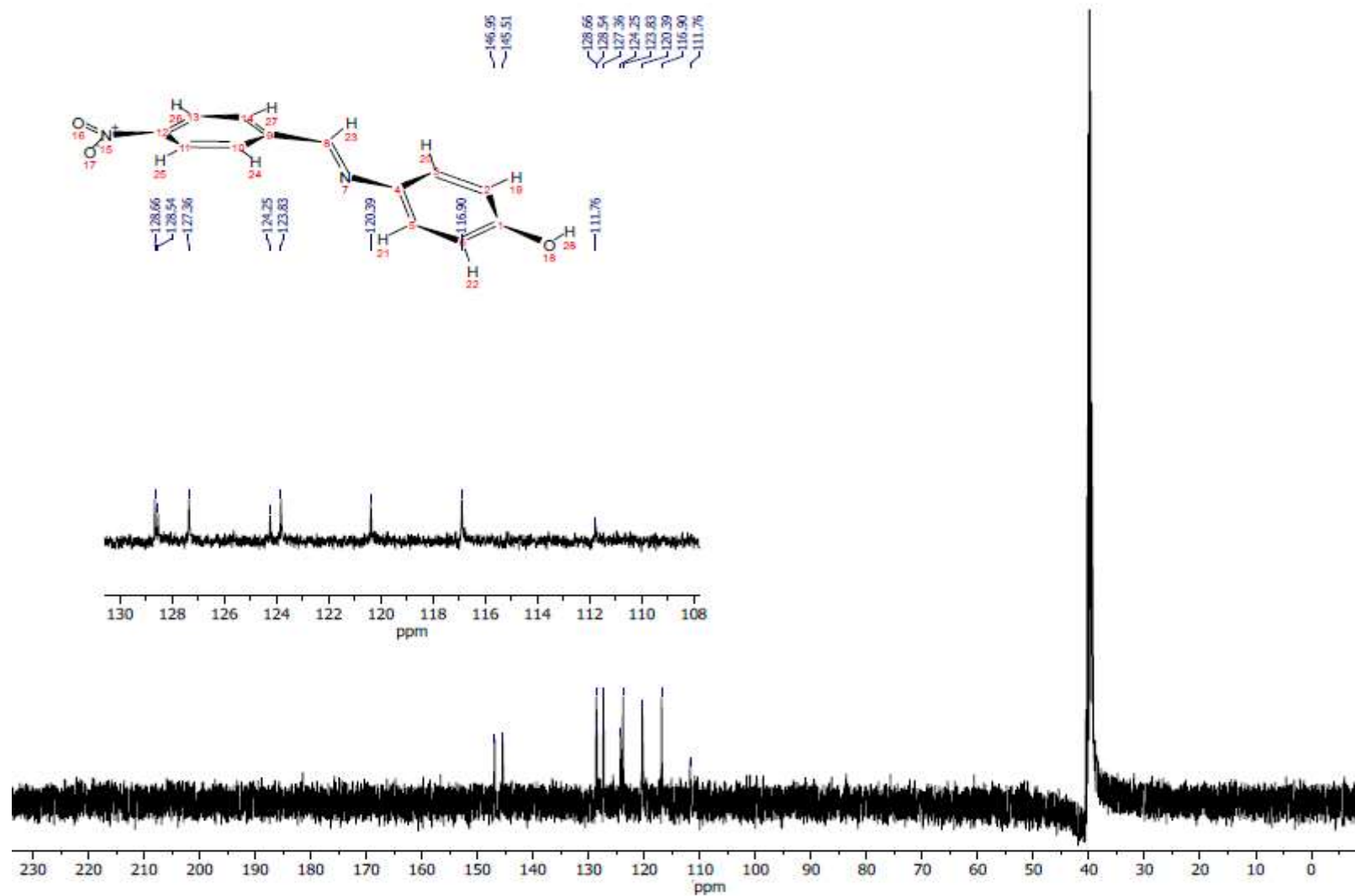

Figure S13:  $^{13}\text{C}$  NMR of Schiff base 5

Supplement: Supplementary file 1 [file antioxidants-07-00113-s001.zip › Figure S13.pdf]

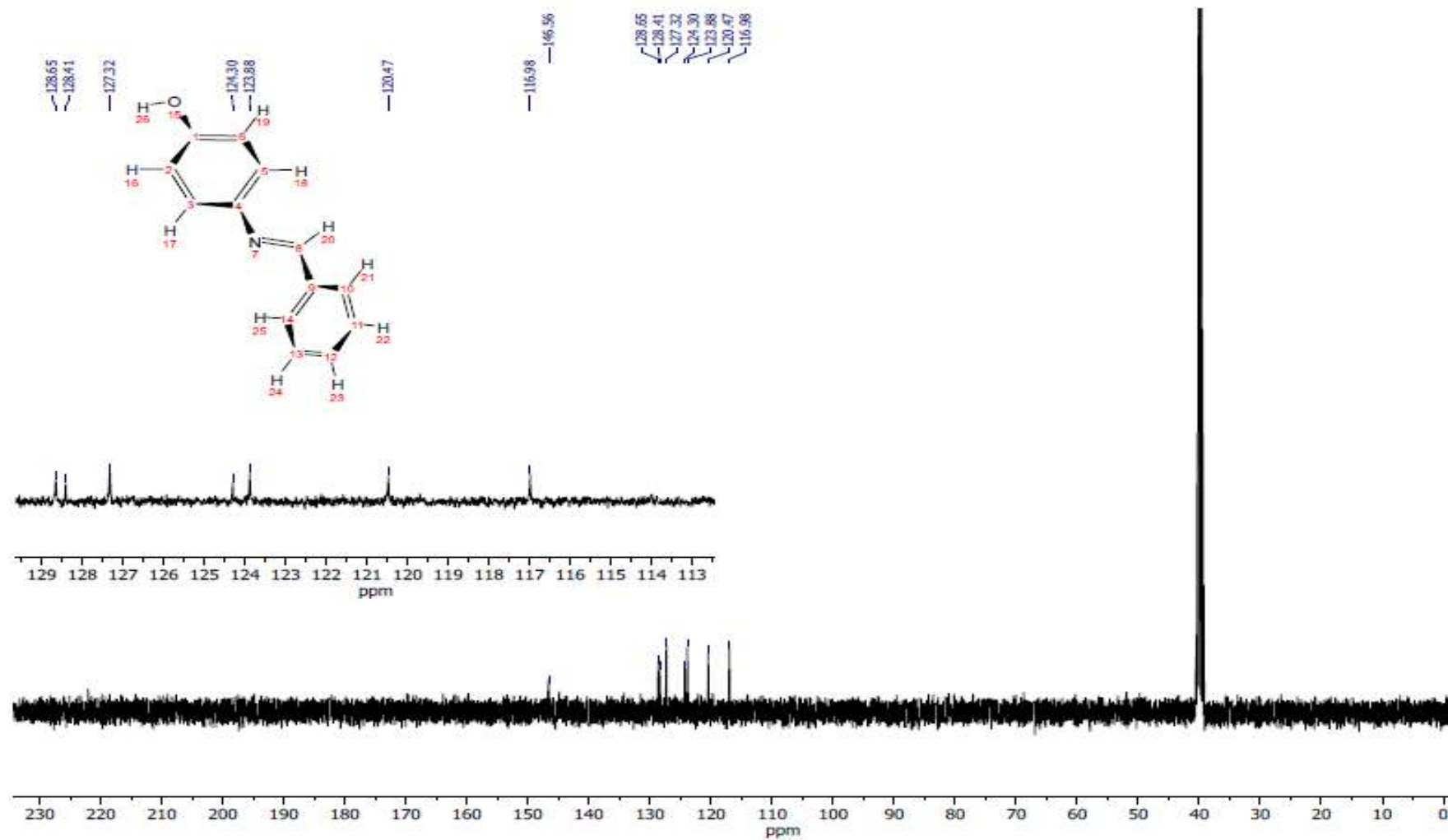

Figure S15:  $^{13}\text{C}$  NMR of Schiff base 6

Supplement: Supplementary file 1 [file antioxidants-07-00113-s001.zip › Figure S15.pdf]

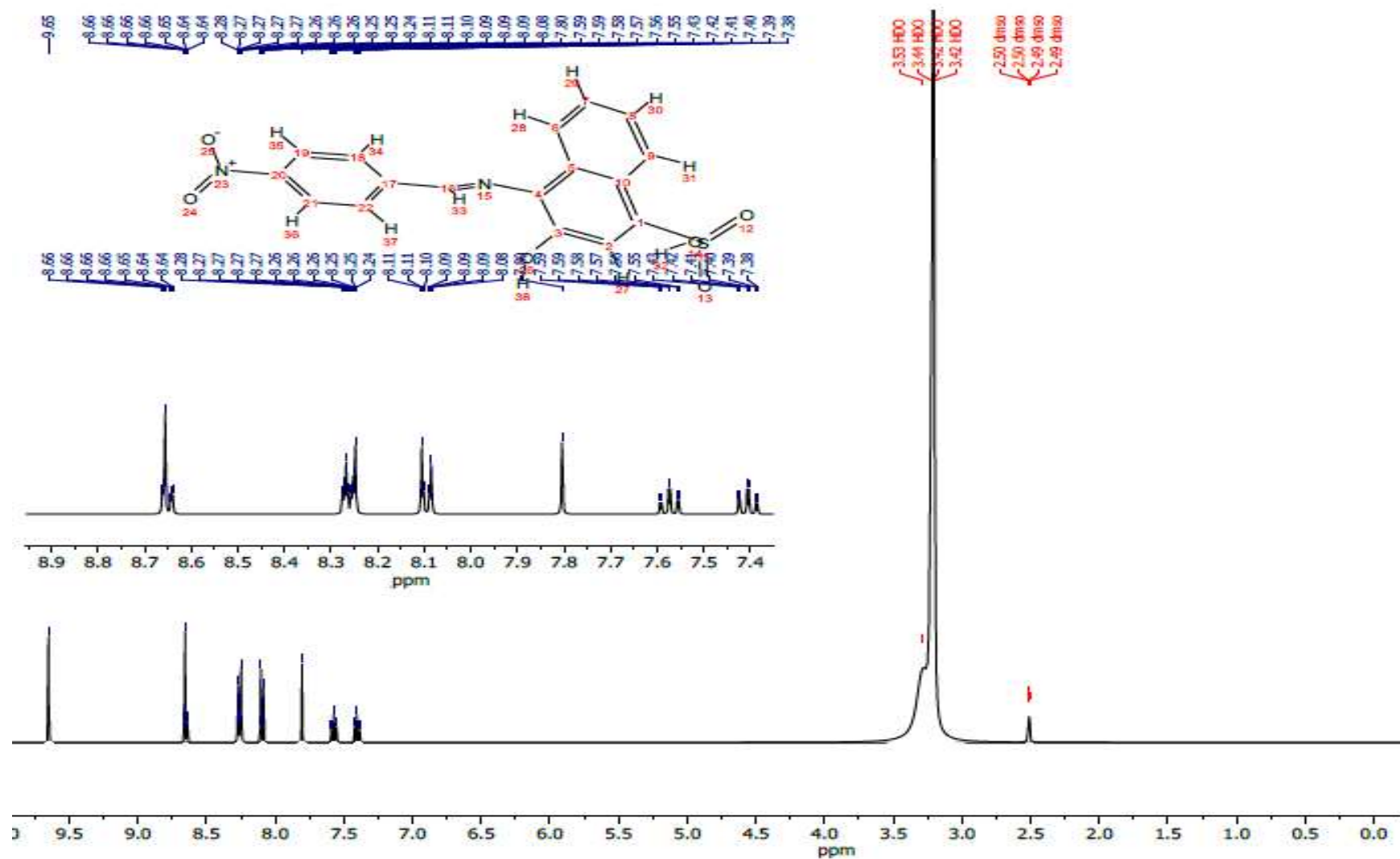

Figure S16:  $^1\text{H}$  NMR of Schiff base 7

Supplement: Supplementary file 1 [file antioxidants-07-00113-s001.zip › Figure S16.pdf]

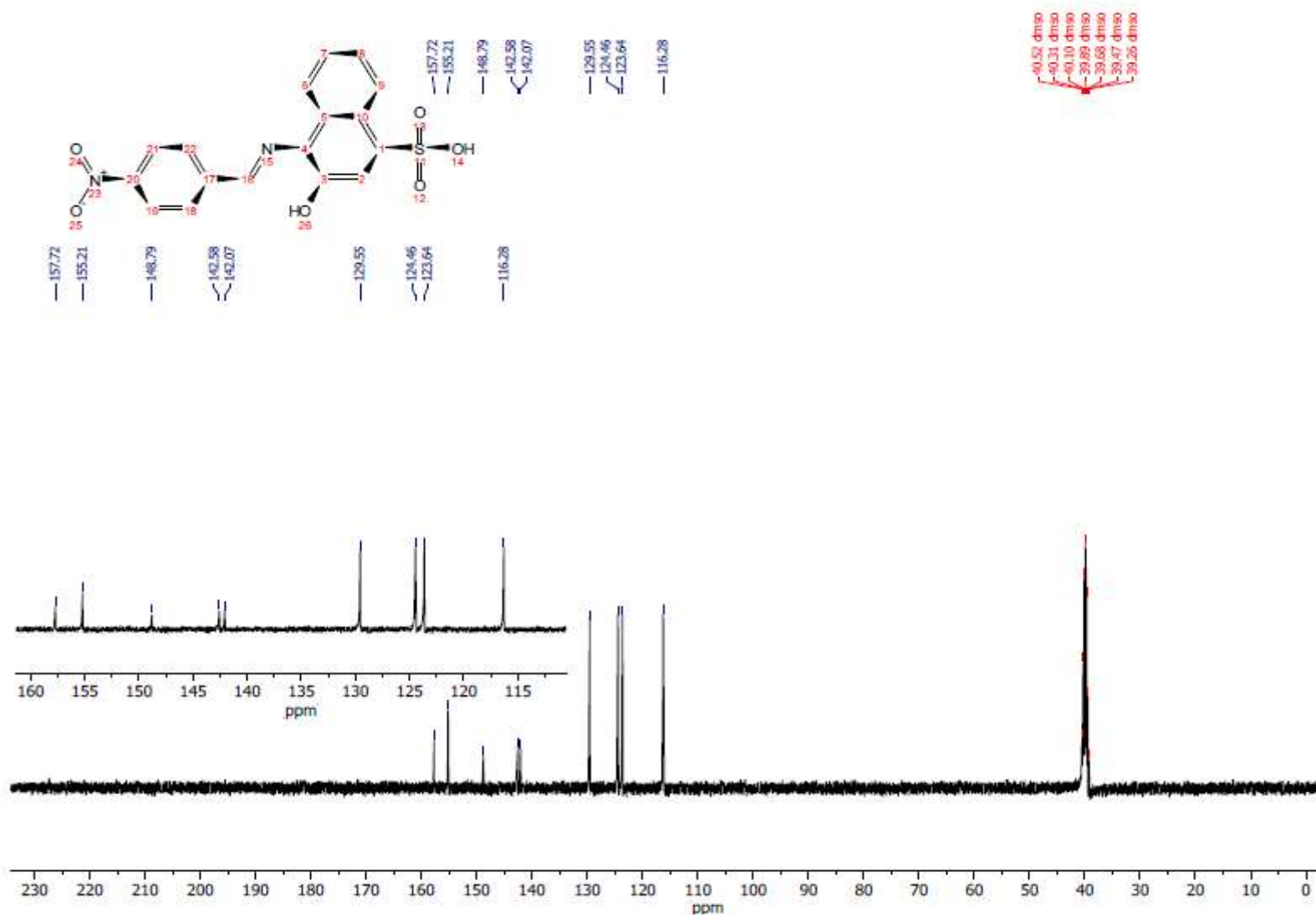

Figure S17: <sup>13</sup>C NMR of Schiff base 7

Supplement: Supplementary file 1 [file antioxidants-07-00113-s001.zip › Figure S17.pdf]

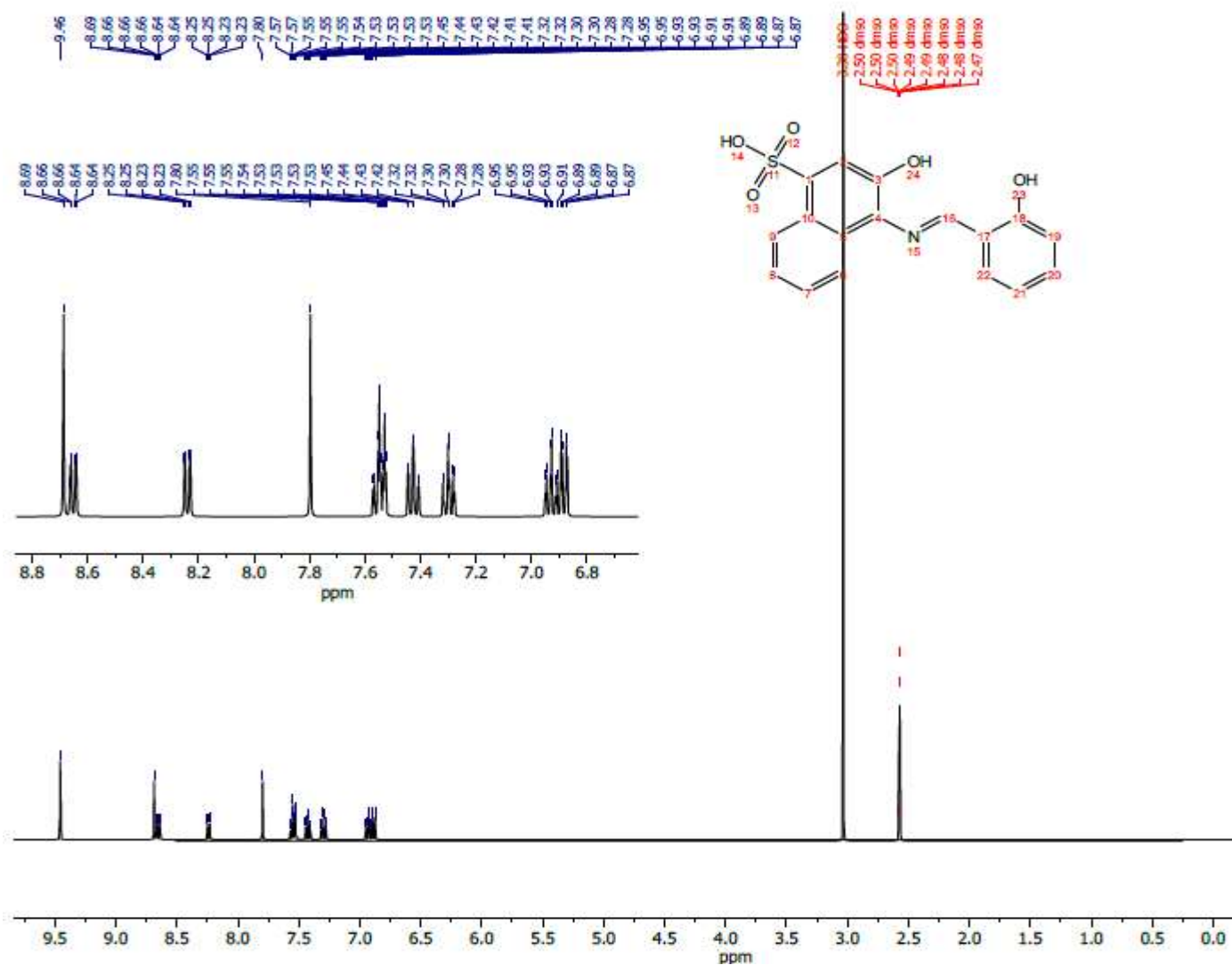

Figure S18:  $^1\text{H}$  NMR of Schiff base 8

Supplement: Supplementary file 1 [file antioxidants-07-00113-s001.zip › Figure S18.pdf]

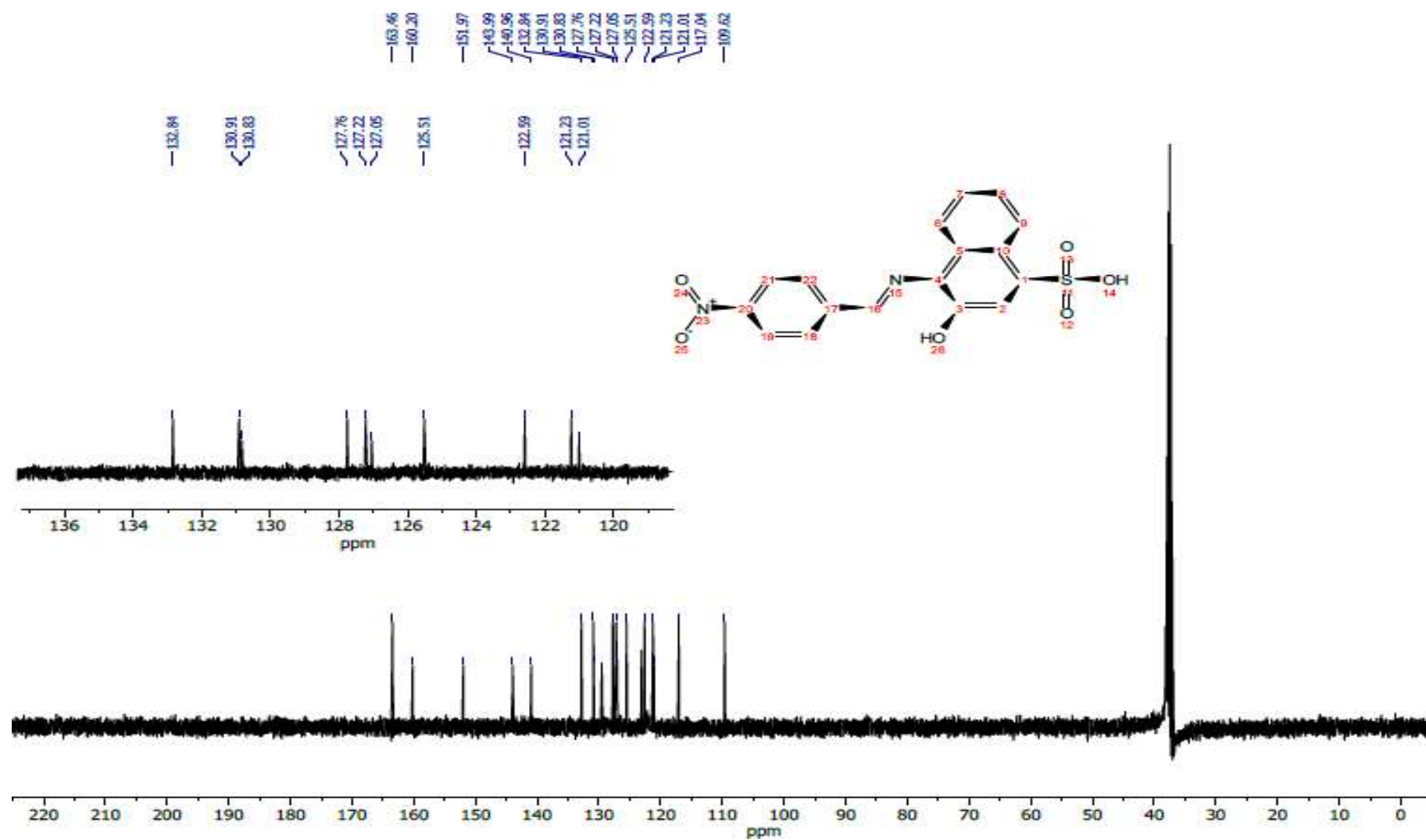

Figure S19: <sup>13</sup>C NMR of Schiff base 8

Supplement: Supplementary file 1 [file antioxidants-07-00113-s001.zip › Figure S19.pdf]

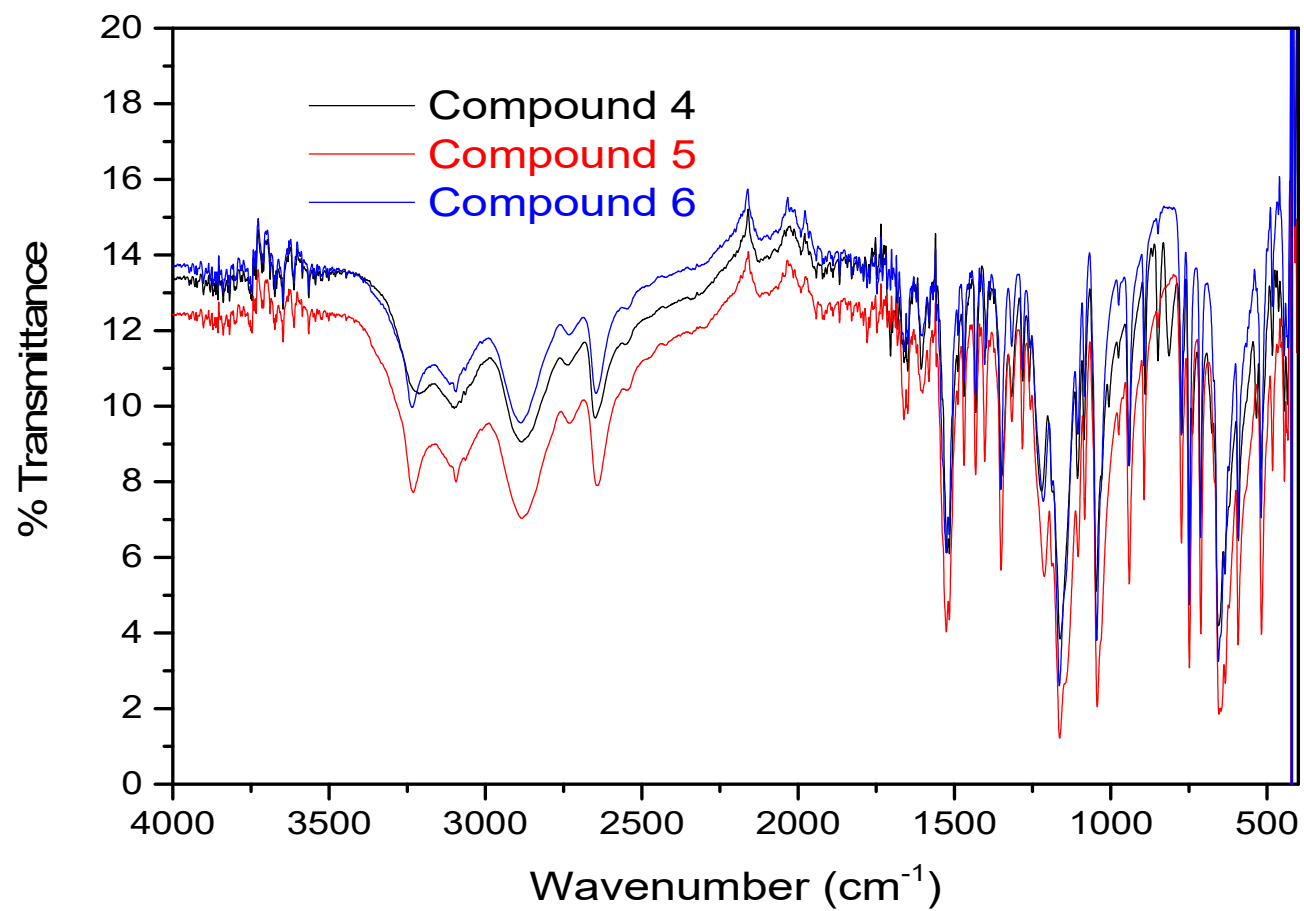

**Figure S2:** FT-IR spectrum of Schiff bases 4-6

Supplement: Supplementary file 1 [file antioxidants-07-00113-s001.zip › Figure S2.pdf]

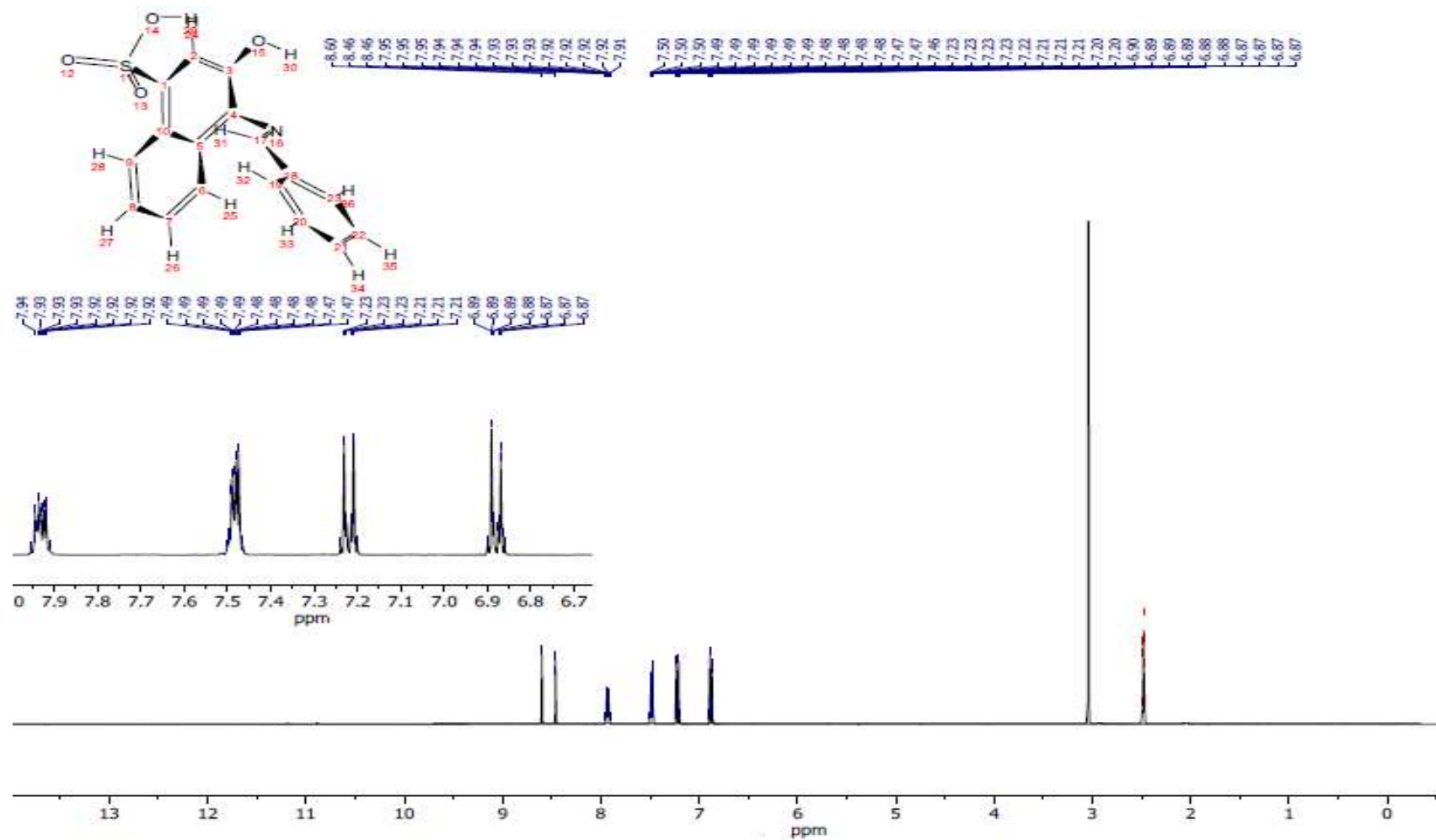

Figure S20:  $^1\text{H}$  NMR of Schiff base 9

Supplement: Supplementary file 1 [file antioxidants-07-00113-s001.zip › Figure S20.pdf]

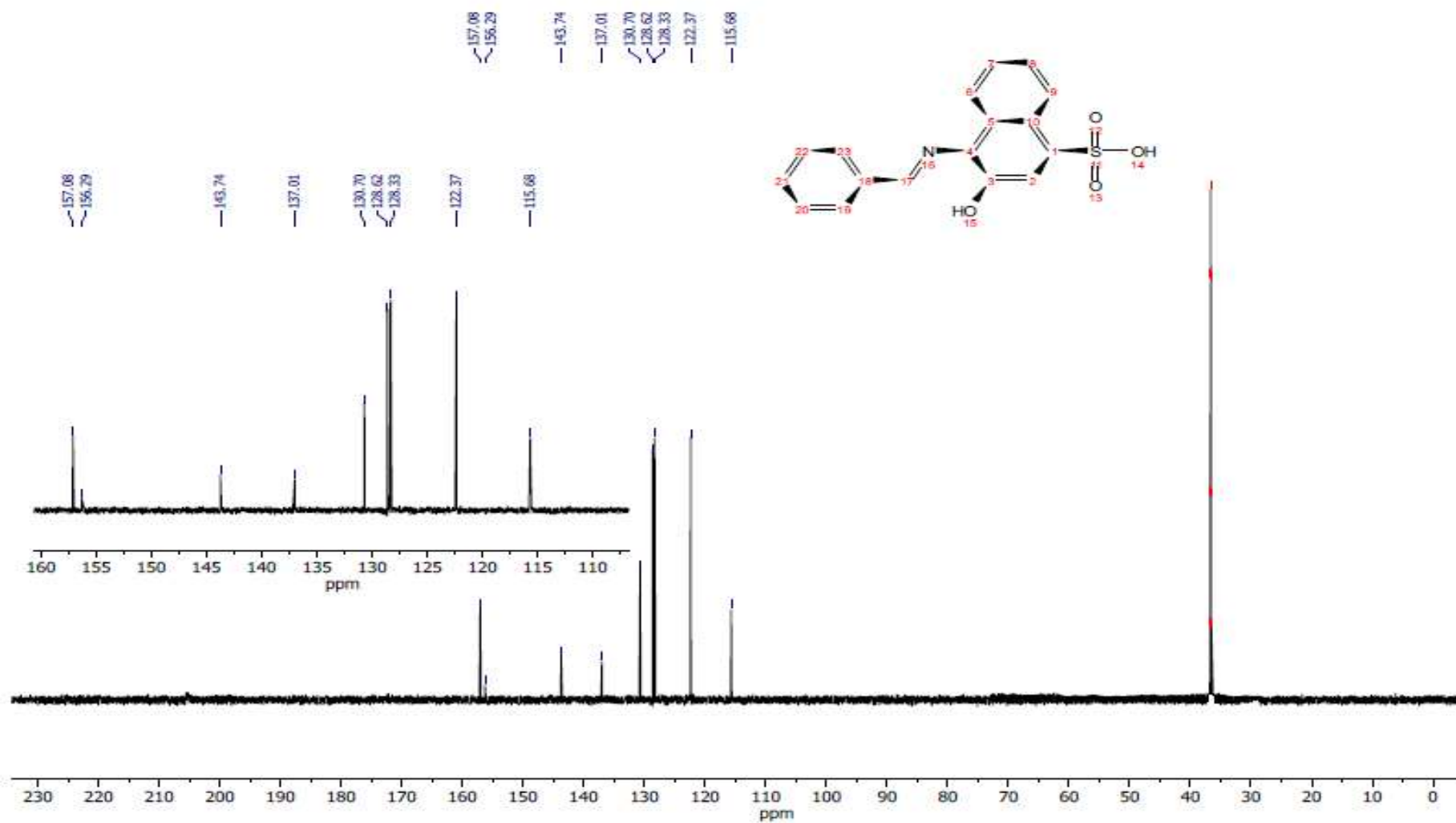

Figure S21:  $^{13}\text{C}$  NMR of Schiff base 9

Supplement: Supplementary file 1 [file antioxidants-07-00113-s001.zip › Figure S21.pdf]

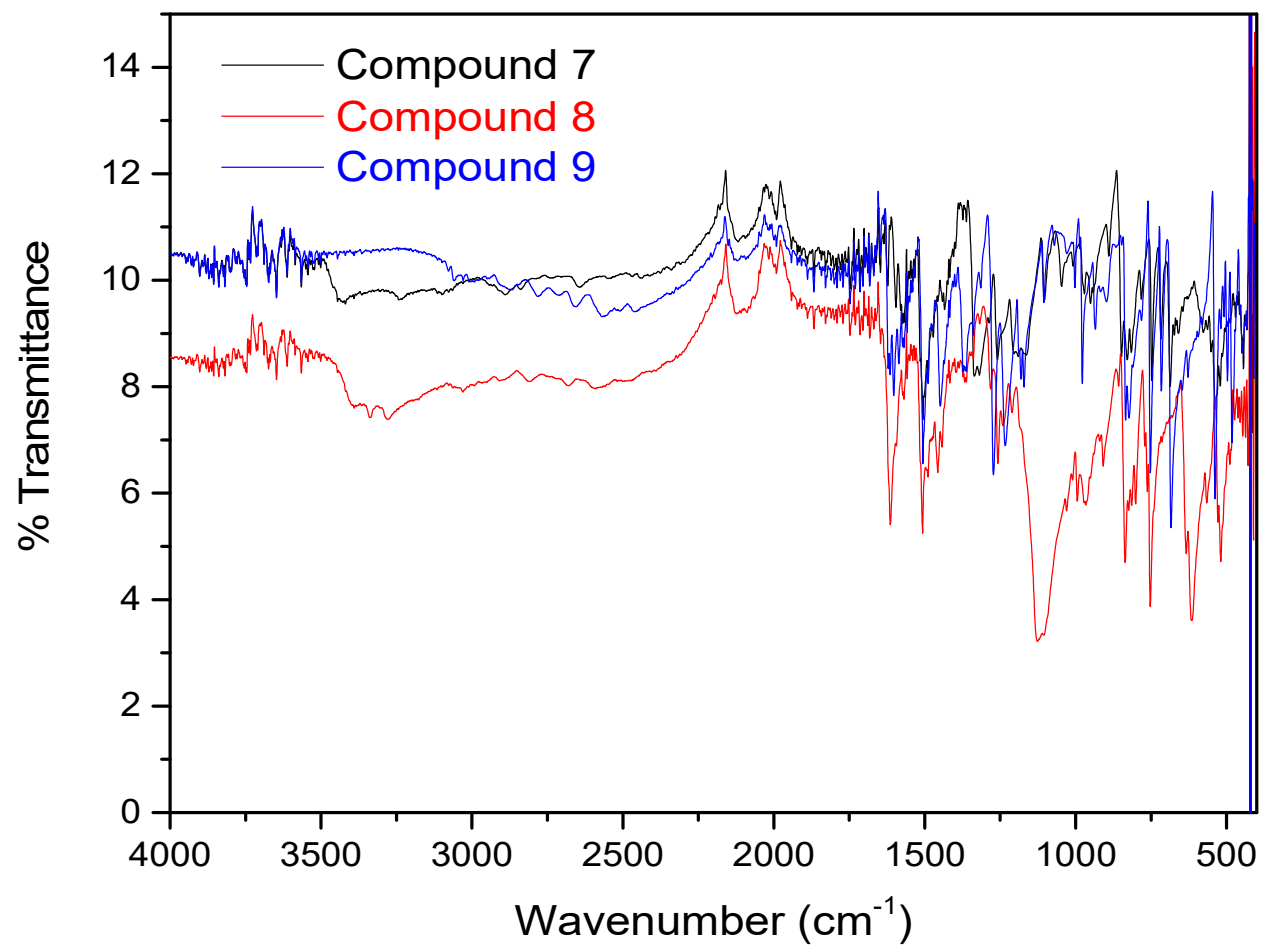

**Figure S3:** FT-IR spectrum of Schiff bases 7-9

Supplement: Supplementary file 1 [file antioxidants-07-00113-s001.zip › Figure S3.pdf]

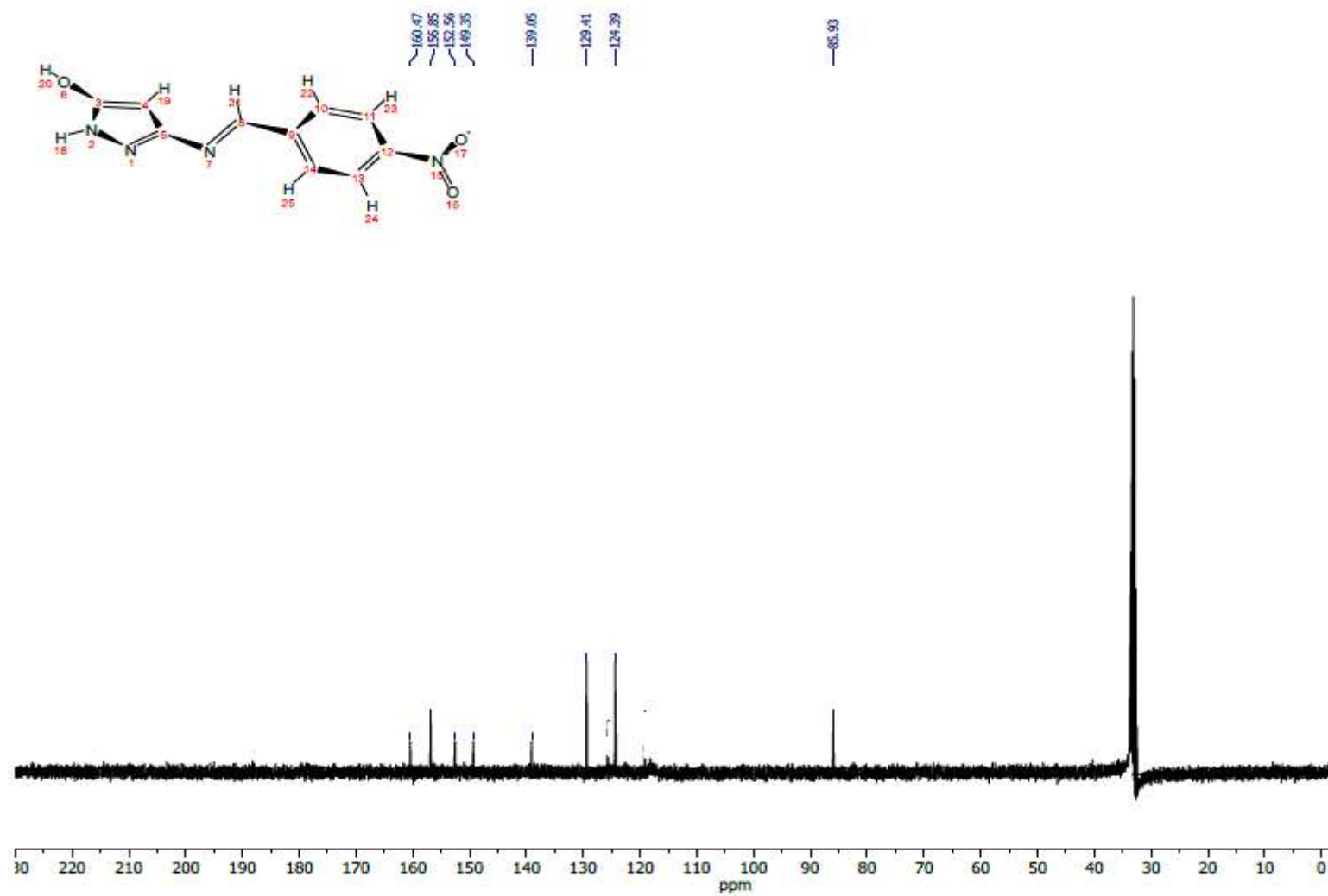

Figure S5:  $^{13}\text{C}$  NMR of Schiff base 1

Supplement: Supplementary file 1 [file antioxidants-07-00113-s001.zip › Figure S5.pdf]

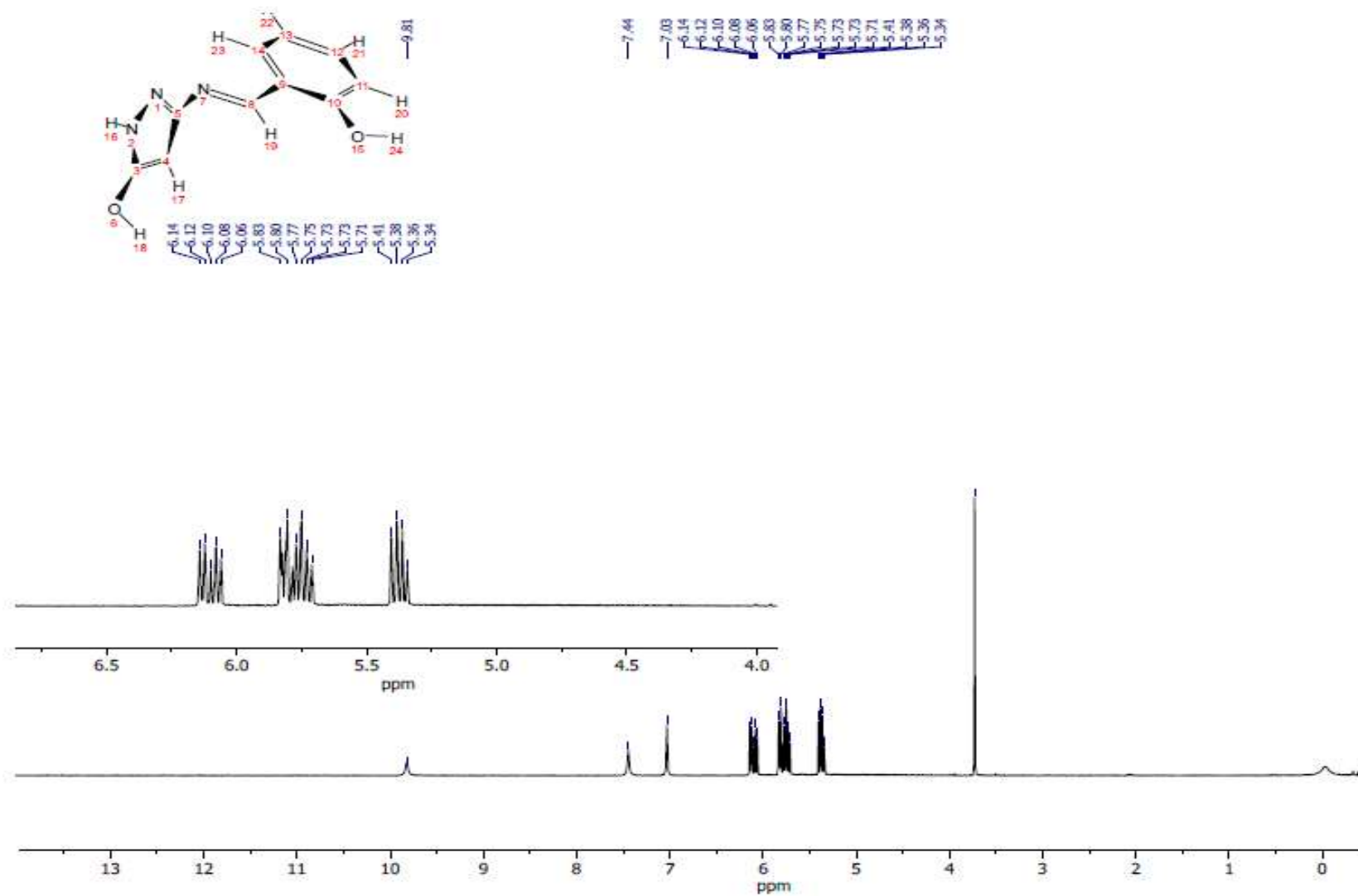

Figure S6:  $^1\text{H}$  NMR of Schiff base 2

Supplement: Supplementary file 1 [file antioxidants-07-00113-s001.zip › Figure S6.pdf]

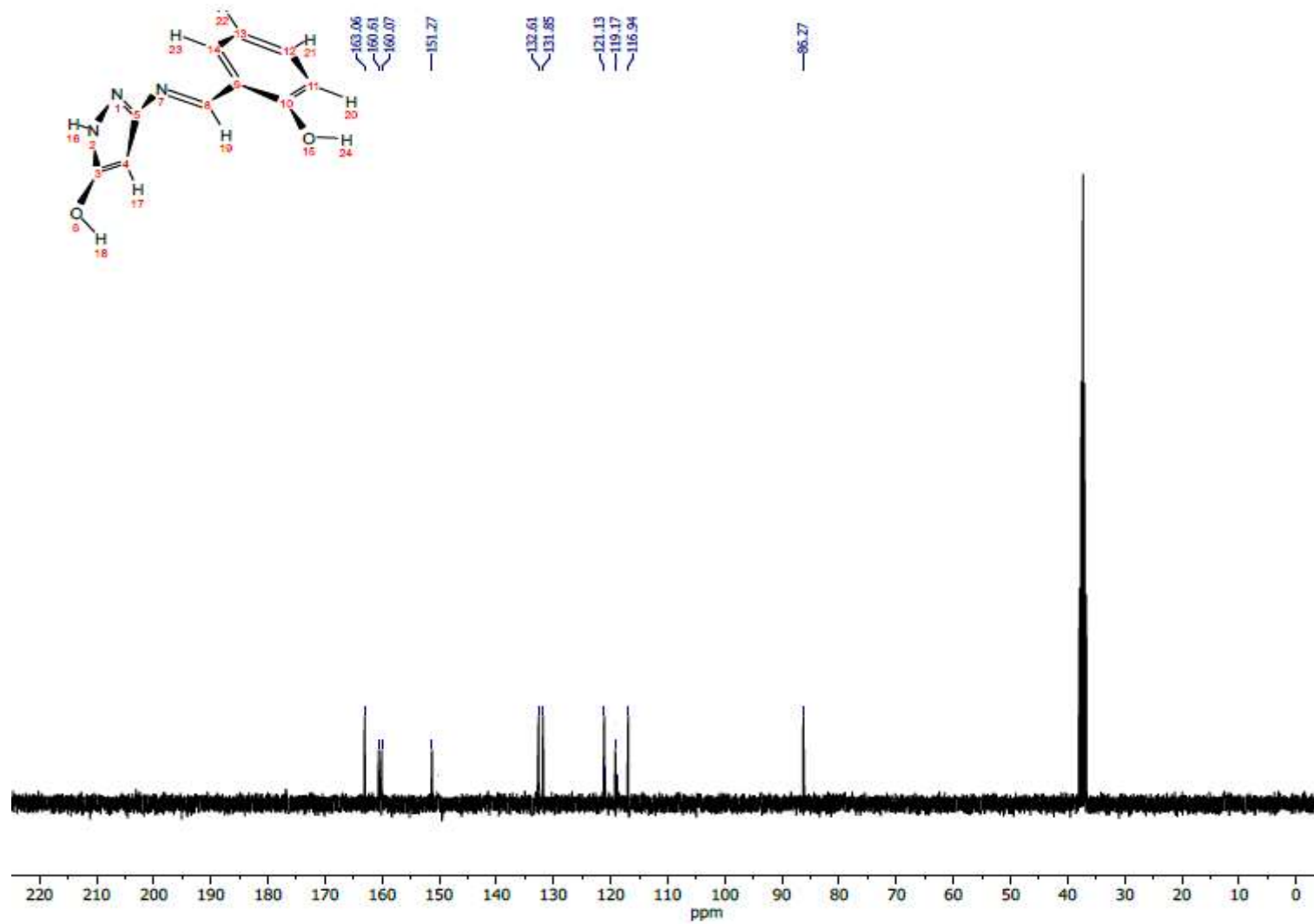

Figure S7:  $^{13}\text{C}$  NMR of Schiff base 2

Supplement: Supplementary file 1 [file antioxidants-07-00113-s001.zip › Figure S7.pdf]

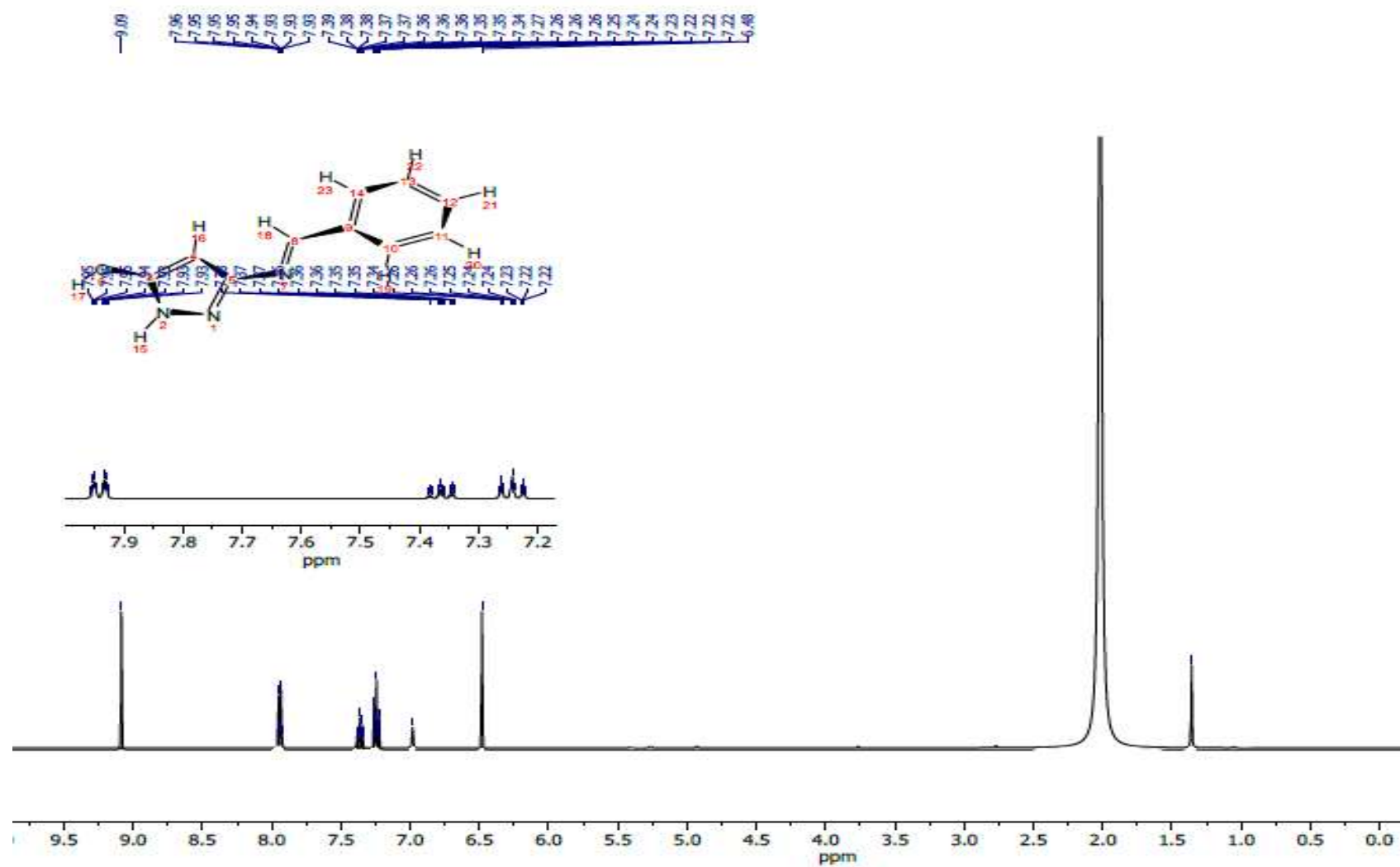

Figure S8:  $^1\text{H}$  NMR of Schiff base 3

Supplement: Supplementary file 1 [file antioxidants-07-00113-s001.zip › Figure S8.pdf]

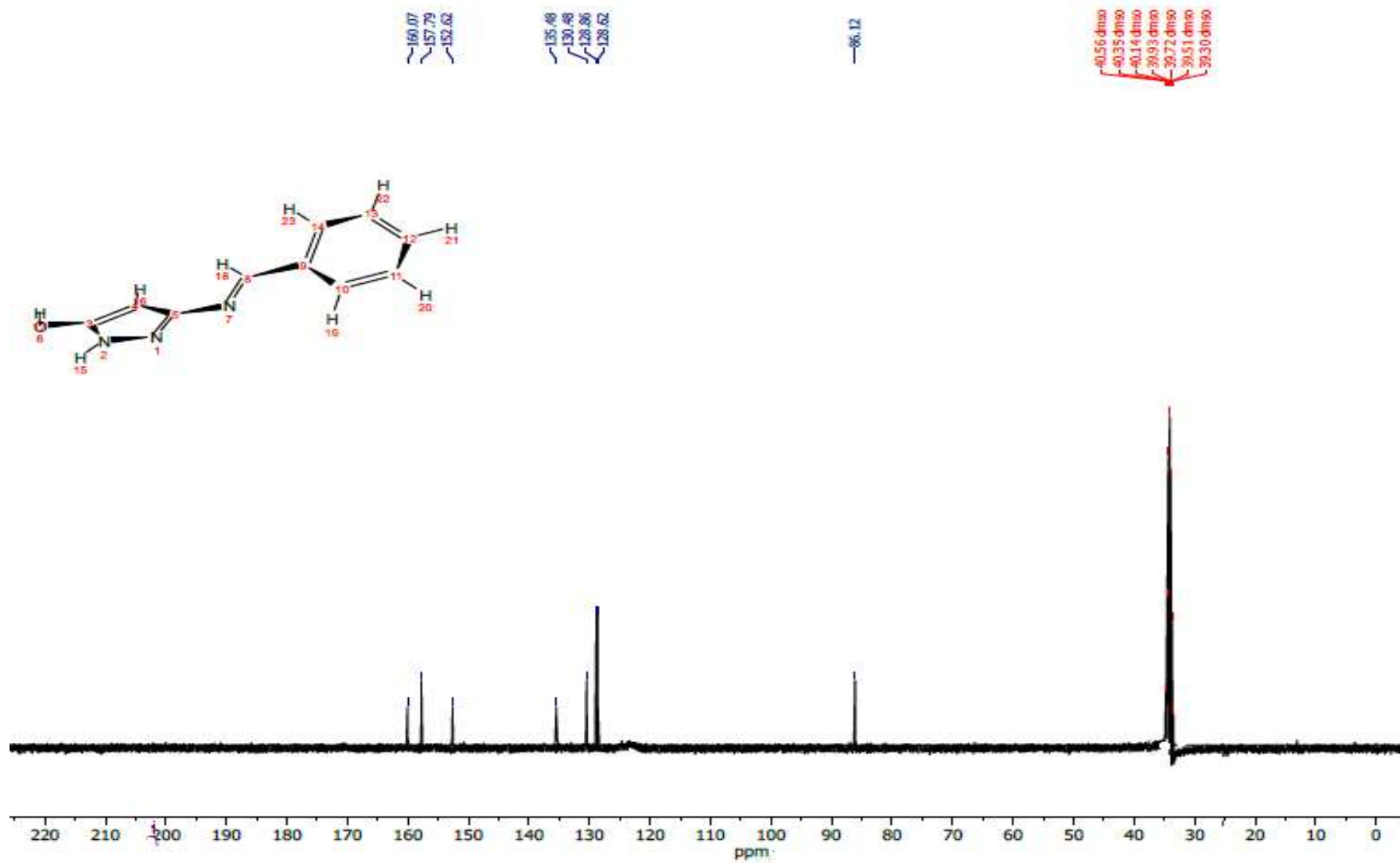

Figure S9:  $^{13}\text{C}$  NMR of Schiff base 3

Supplement: Supplementary file 1 [file antioxidants-07-00113-s001.zip › Figure S9.pdf]

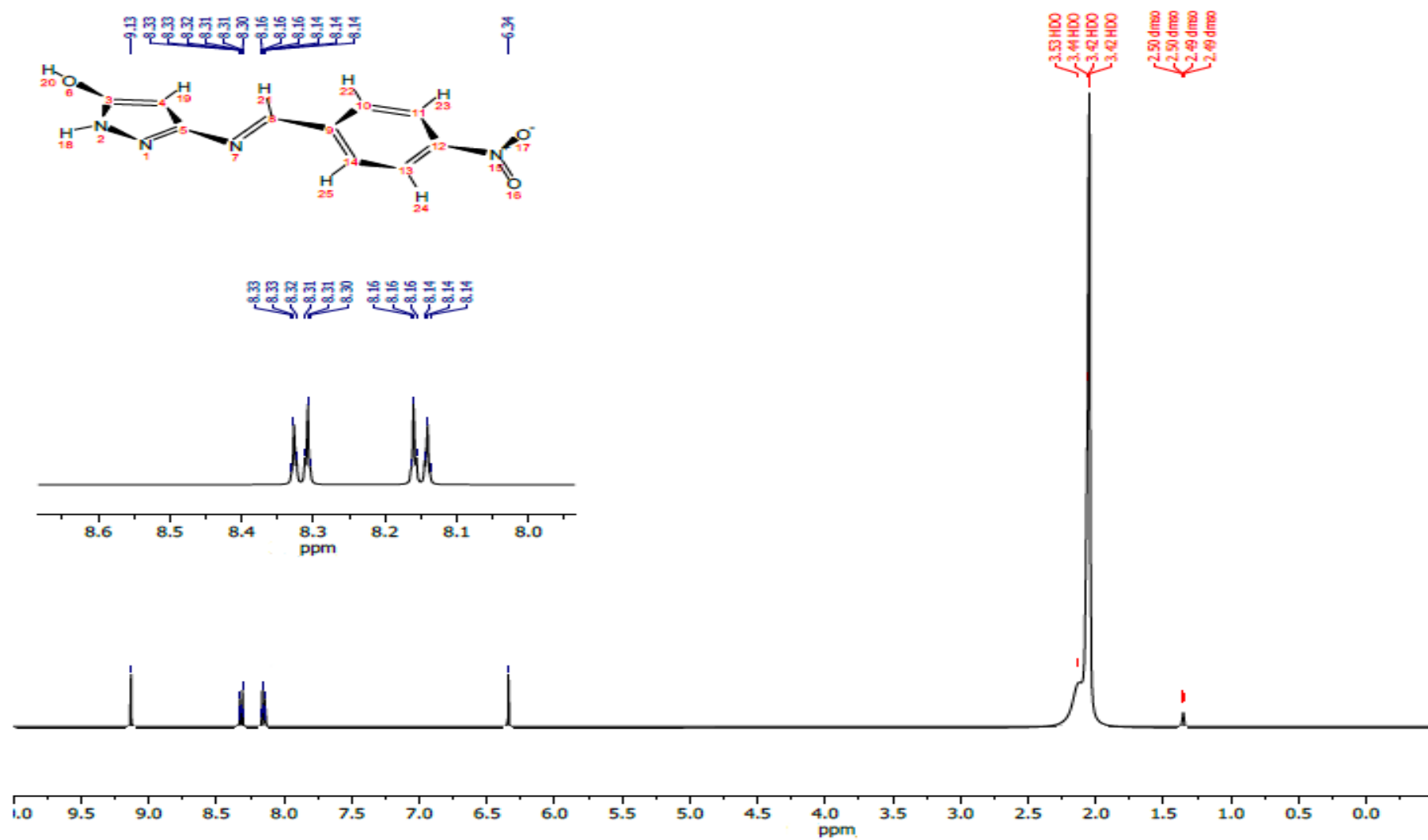

Figure S4:  $^1\text{H}$  NMR of Schiff base 1

Supplement: Supplementary file 1 [file antioxidants-07-00113-s001.zip › Figure S4.pdf]
