# Supplementary material for: Investigation into the Thermal Response and Pharmacological Activity of Substituted Schiff Bases on α-Amylase and α-Glucosidase
Source: Antioxidants (Basel). 2018 Aug 28;7(9):113. doi: 10.3390/antiox7090113 (PMC6162693; doi:10.3390/antiox7090113)
Supplement: Supplementary file 1 [file Figure S14.pdf › Figure S14.pdf]

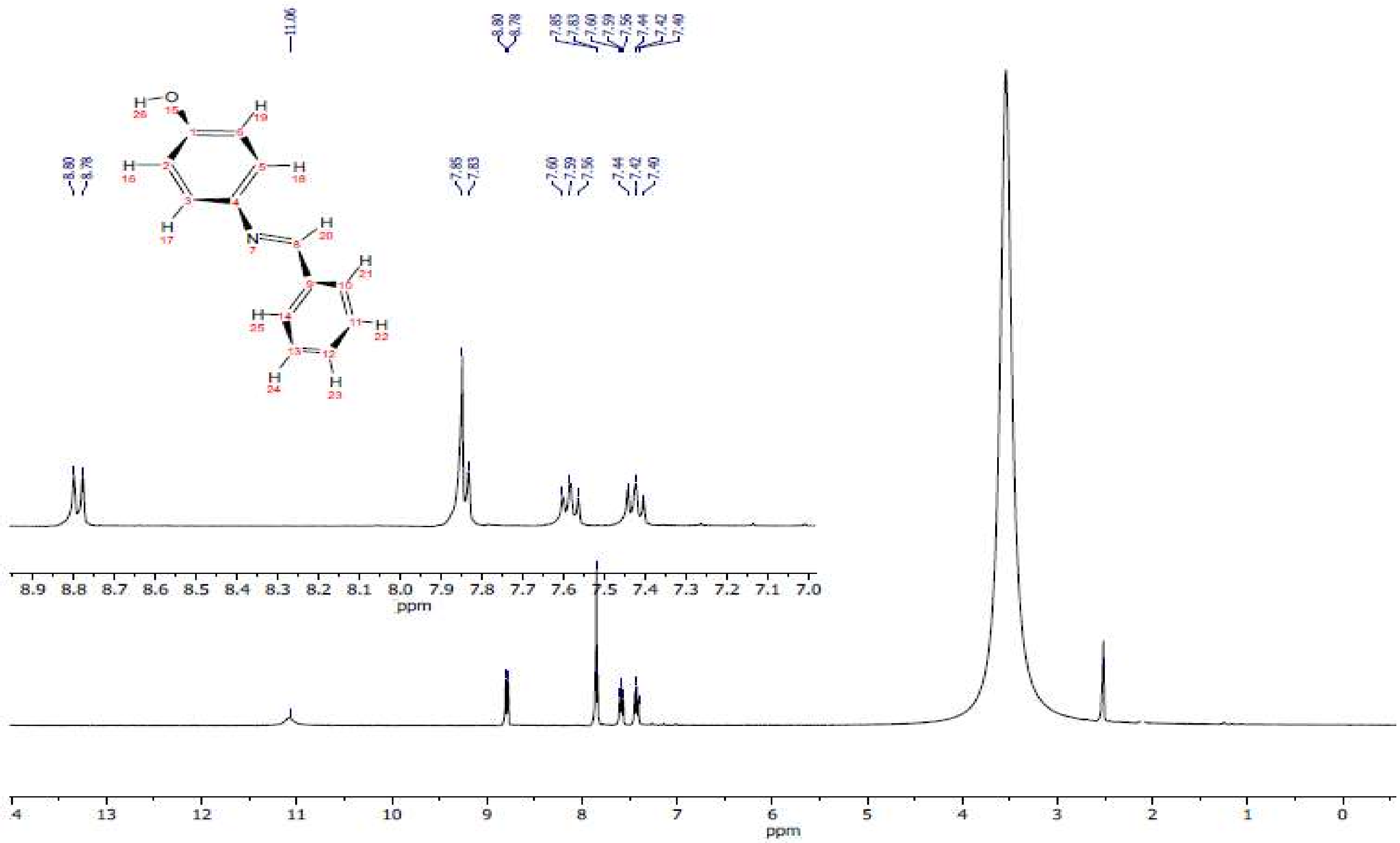

Figure S14: ^{1}H NMR of Schiff base 6
